# A genome-scale metabolic model of a globally disseminated hyperinvasive M1 strain of *Streptococcus pyogenes*

**DOI:** 10.1128/msystems.00736-24

**Published:** 2024-08-19

**Authors:** Yujiro Hirose, Daniel C. Zielinski, Saugat Poudel, Kevin Rychel, Jonathon L. Baker, Yoshihiro Toya, Masaya Yamaguchi, Almut Heinken, Ines Thiele, Shigetada Kawabata, Bernhard O. Palsson, Victor Nizet

**Affiliations:** 1 Department of Microbiology, Osaka University Graduate School of Dentistry, Suita, Osaka, Japan; 2 Department of Pediatrics, University of California at San Diego School of Medicine, La Jolla, California, USA; 3 Department of Bioengineering, University of California San Diego, La Jolla, California, USA; 4 Genomic Medicine Group, J. Craig Venter Institute, La Jolla, California, USA; 5 Department of Oral Rehabilitation & Biosciences, OHSU School of Dentistry, Portland, Oregon, USA; 6 Department of Bioinformatic Engineering, Graduate School of Information Science and Technology, Osaka University, Suita, Osaka, Japan; 7 Bioinformatics Research Unit, Graduate School of Dentistry, Osaka University, Suita, Osaka, Japan; 8 Bioinformatics Center, Research Institute for Microbial Diseases, Osaka University, Suita, Osaka, Japan; 9 Center for Infectious Diseases Education and Research, Osaka University, Suita, Osaka, Japan; 10 School of Medicine, National University of Galway, Galway, Ireland; 11 Ryan Institute, University of Galway, Galway, Ireland; 12 Inserm UMRS 1256 NGERE, University of Lorraine, Nancy, France; 13 Division of Microbiology, National University of Galway, Galway, Ireland; 14 APC Microbiome Ireland, Cork, Ireland; 15 Skaggs School of Pharmaceutical Sciences, University of California at San Diego, La Jolla, California, USA; Indian Institute of Technology, Mandi, India

**Keywords:** *Streptococcus pyogenes*, metabolic modeling, genome-scale model, auxotrophy, carbon sources, essential gene

## Abstract

*Streptococcus pyogenes* is responsible for a range of diseases in humans contributing significantly to morbidity and mortality. Among more than 200 serotypes of *S. pyogenes*, serotype M1 strains hold the greatest clinical relevance due to their high prevalence in severe human infections. To enhance our understanding of pathogenesis and discovery of potential therapeutic approaches, we have developed the first genome-scale metabolic model (GEM) for a serotype M1 *S. pyogenes* strain, which we name iYH543. The curation of iYH543 involved cross-referencing a draft GEM of *S. pyogenes* serotype M1 from the AGORA2 database with gene essentiality and autotrophy data obtained from transposon mutagenesis-based and growth screens. We achieved a 92.6% (503/543 genes) accuracy in predicting gene essentiality and a 95% (19/20 amino acids) accuracy in predicting amino acid auxotrophy. Additionally, Biolog Phenotype microarrays were employed to examine the growth phenotypes of *S. pyogenes,* which further contributed to the refinement of iYH543. Notably, iYH543 demonstrated 88% accuracy (168/190 carbon sources) in predicting growth on various sole carbon sources. Discrepancies observed between iYH543 and the actual behavior of living *S. pyogenes* highlighted areas of uncertainty in the current understanding of *S. pyogenes* metabolism. iYH543 offers novel insights and hypotheses that can guide future research efforts and ultimately inform novel therapeutic strategies.

**IMPORTANCE:**

Genome-scale models (GEMs) play a crucial role in investigating bacterial metabolism, predicting the effects of inhibiting specific metabolic genes and pathways, and aiding in the identification of potential drug targets. Here, we have developed the first GEM for the *S. pyogenes* highly virulent serotype, M1, which we name iYH543. The iYH543 achieved high accuracy in predicting gene essentiality. We also show that the knowledge obtained by substituting actual measurement values for iYH543 helps us gain insights that connect metabolism and virulence. iYH543 will serve as a useful tool for rational drug design targeting *S. pyogenes* metabolism and computational screening to investigate the interplay between inhibiting virulence factor synthesis and growth.

## INTRODUCTION

*Streptococcus pyogenes* is responsible for a wide range of diseases in humans, causing over 700 million infections and at least 517,000 deaths worldwide annually (1). Among the 200+ serotypes of *S. pyogenes*, serotype M1 is the most frequently isolated from streptococcal pharyngitis (2) and invasive diseases worldwide (3), and it is extensively studied to assess virulence mechanisms during infection (4–7). On the other hand, serotype M49 is commonly used by researchers due to the ease of genetic manipulation, despite generally possessing weaker disease phenotypes in infection models (8). Studies on particularly pathogenic *S. pyogenes* strains*,* including those of serotype M1, have revealed the significance of metabolic and transporter genes as virulence factors in necrotizing myositis (4) and pharyngitis (5). These genes, thus, hold potential as novel therapeutic targets. Genome-scale models (GEMs) play a crucial role in investigating bacterial metabolism, predicting the effects of inhibiting specific metabolic genes and pathways, and aiding in the identification of potential drug targets (9, 10). Although a GEM for *S. pyogenes* serotype M49 (strain 591) was previously developed by Levering et al. (11), there is currently no GEM available for predicting metabolism and identifying drug targets specifically for strains of the more virulent and clinically relevant M1 serotype.

In this study, we developed and curated a highly accurate GEM for *S. pyogenes* serotype M1. To construct this model, we leveraged a draft GEM of *S. pyogenes* serotype M1 (strain SF370) from the AGORA2 computational resource (12), which we manually curated using information from various databases including BiGG (13), VMH (14), BioCyc (15), KEGG (16), and EC number information from EggNOG (17). We carefully examined newly generated experimental data (Biolog growth data) and external experimental data, including conditionally defined media (CDM) growth and transposon mutagenesis-based gene essentiality of *S. pyogenes* M1 (strain 5448) (18), to resolve discrepancies within the initial draft GEM derived from AGORA2. As a result, we significantly improved the accuracy of gene essentiality predictions from 73.6% (351/477 genes) in the draft GEM to 92.6% (514/543 genes) in the final curated GEM, iYH543. This accuracy surpasses the previously reported *S. pyogenes* GEM, which achieved 76.6% accuracy. Furthermore, we demonstrate that iYH543 can be effectively used to investigate the metabolic behavior of *S. pyogenes* on the genome scale using flux balance analysis (FBA) simulation (19). This provides a highly accurate platform to explore the metabolic behavior of *S. pyogenes* under diverse growth conditions, which may aid in the identification of novel therapeutic leads.

## RESULTS

### Overview of the AGORA2-derived draft GEM of *S. pyogenes* serotype M1 and iYH543

**Fig 1:**
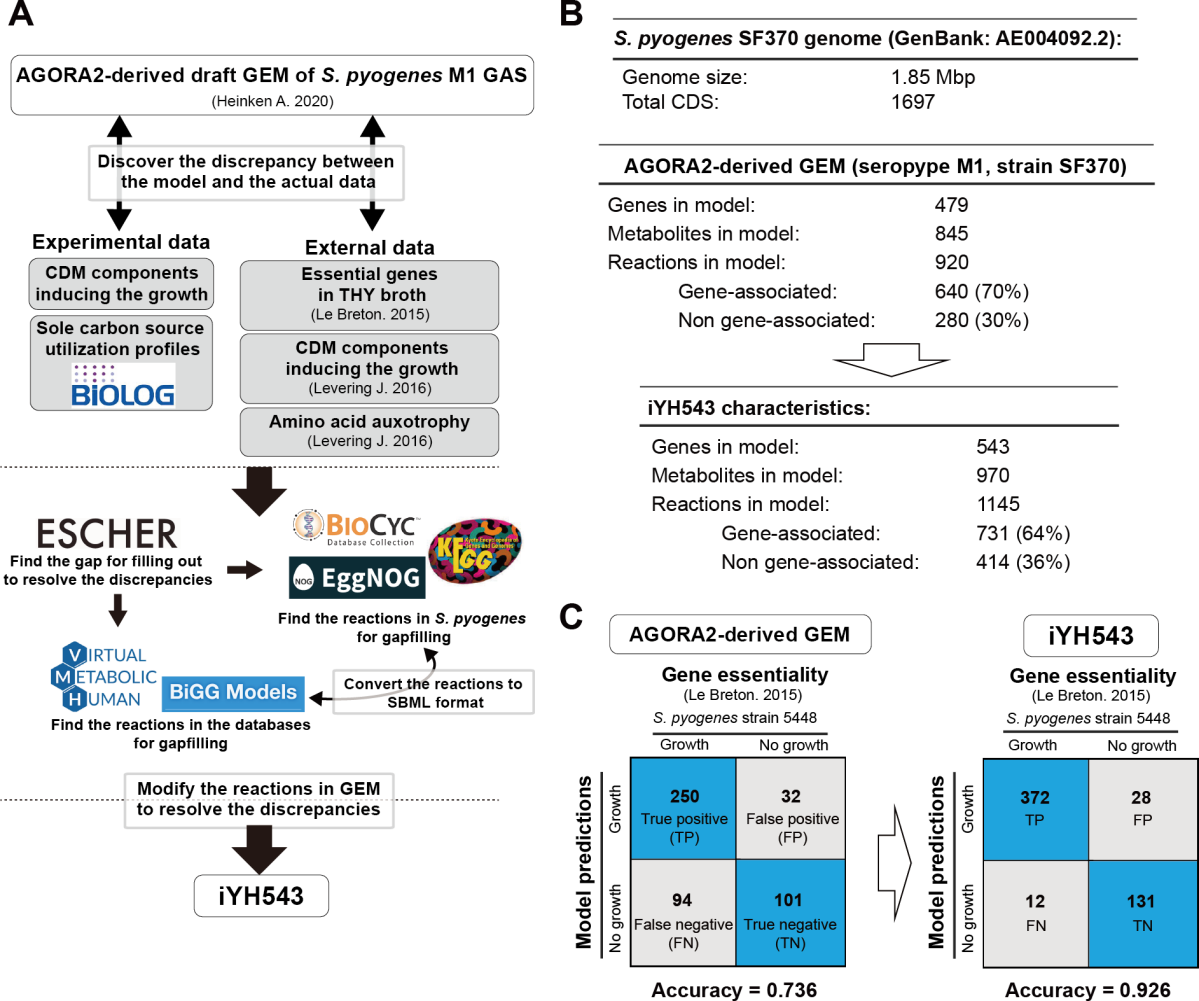
Curation strategy of the AGORA2-derived genome-scale metabolic model (draft GEM) of *S. pyogenes* M1 GAS (strain SF370) to produce iYH543. (**A**) Schematic of the network reconstruction strategy for iYH543. The experimental data from this study and previously published data from other studies were compared with the results of the simulation in the AGORA2-derived draft GEM. Based on the identified discrepancies, the model was curated and reconstructed to produce iYH543. (**B**) Attributes of the AGORA2-derived draft GEM and iYH543. Details regarding the genes, metabolites, and reactions included in iYH543 are listed in Table S2. (**C**) Accuracy of essentiality predictions in the AGORA2-derived draft GEM and iYH543. Transposon mutagenesis-based gene essentiality in *S. pyogenes* strain 5448 (serotype M1) was used for validation. AGORA2 (12). Transposon mutagenesis-based gene essentiality (18). CDM components and amino acid auxotrophy (11). SBML, Systems Biology Markup Language. Accuracy: (TP + TN)/all genes.

In this study, we present iYH543, a curated GEM of *S. pyogenes* serotype M1. The strategy used to produce iYH543 is summarized in Fig. 1. We started with a draft GEM of *S. pyogenes* serotype M1 strain SF370 derived from AGORA2, which contained 479 genes, 845 metabolites, and 920 reactions. To incorporate known gene essentiality (18) and auxotrophy (4, 11) information, we modified the draft GEM by adding 239 reactions, modifying 112 gene–protein–reaction (GPR) rules, deleting three reactions, and adjusting the biomass reaction (bio1; the objective of our flux balance analysis). Detailed modifications are provided in Table S1. To validate and further refine the draft GEM, we assessed sole carbon source utilization profiles and growth in various components of a CDM. Discrepancies between the experimental data and draft GEM were visualized using Escher (20) and addressed by referencing information from BiGG (13), VMH (14), BioCyc (15), KEGG (16), and EggNOG (17) (Fig. 1A). The manual curation processes for iYH543 are explained in subsequent sections. The final curated GEM, iYH543, consisted of 543 genes, 970 metabolites, and 1,145 reactions (Fig. 1B). The details of all reactions, metabolites, and GPRs in iYH543 can be found in Table S2 or DataS1_iYH543.json at http://www.dent.osaka-u.ac.jp/wp-content/uploads/2024/03/Code_Model_Files_Hirose_et_al.zip. Computing the essential genes in each model and comparing results with a published genome-scale mutant screen (18), the draft GEM achieved 73.6% accuracy, while iYH543 had a significantly improved accuracy of 92.6% (Fig. 1C).

**Fig 2:**
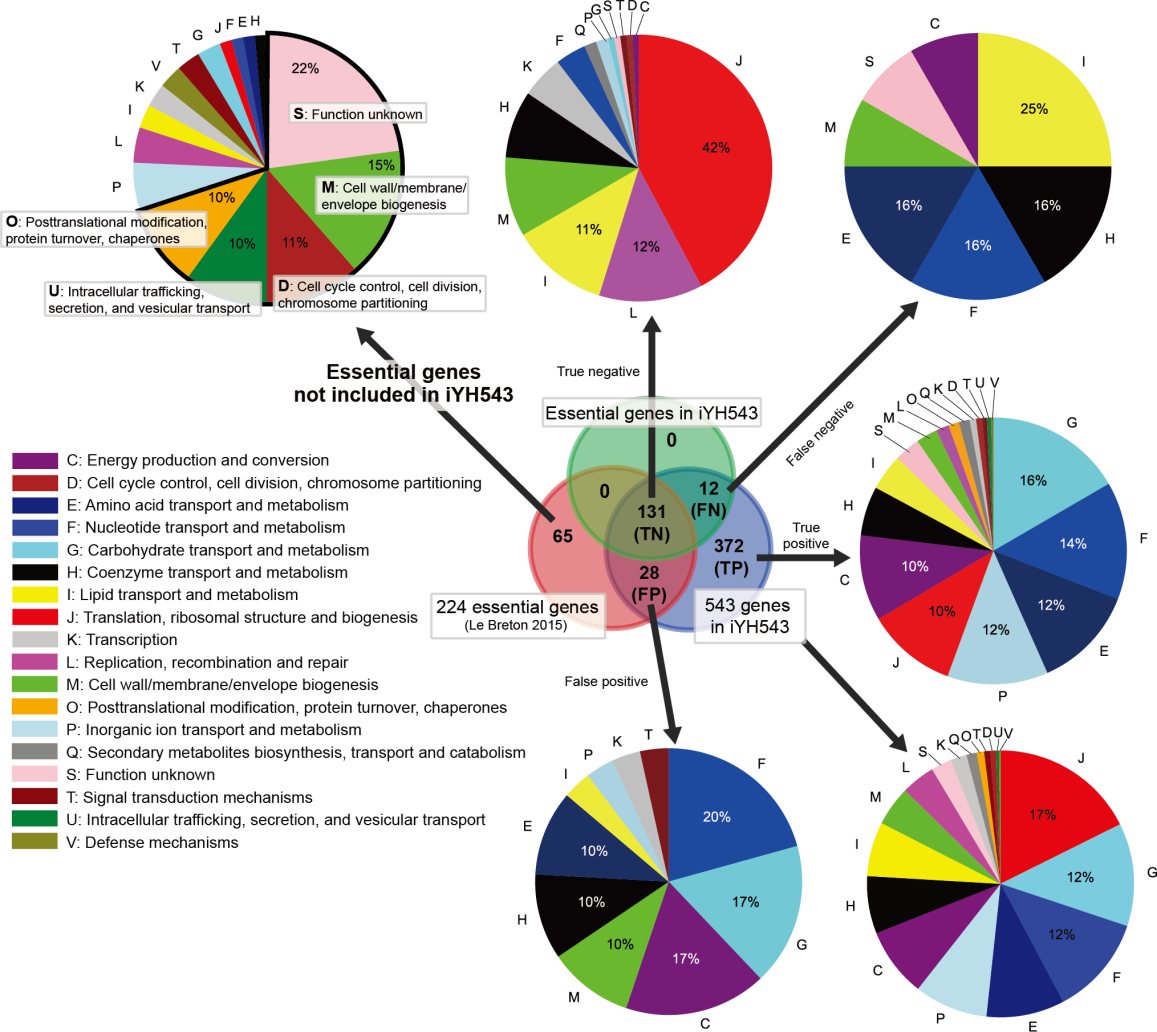
COG categories represented in the true positive (TP), true negative (TN), false positive (FP), and false negative (FN) groups of genes, as well as the 65 essential genes not in iYH543. COGs are colored for each function. Sixty-five genes out of 224 essential genes, which were not included in iYH543, mainly having COG categories of S (function unknown), M (cell wall/membrane/envelope biogenesis), D (cell cycle control, cell division, and chromosome partitioning), U (intracellular trafficking, secretion, and vesicular transport), and O (posttranslational modification, protein turnover, and chaperones). Transposon mutagenesis-based gene essentiality (18).

Le Breton et al. conducted a study on the essentiality of 227 genes for *S. pyogenes* strain 5448, which belongs to the M1 serotype and is closely related to strain SF370. The study was performed under standard laboratory conditions (37°C, 5% CO_2_) using Todd–Hewitt yeast (THY) medium (18). Among the 227 essential genes identified in strain 5448, strain SF370 possesses 224 corresponding orthologs, as determined through bidirectional BLAST hits. Notably, the three unique proteins in strain 5448 are not metabolic enzymes or transporters. Of the 224 orthologous genes, 65 are not included in iYH543 (Fig. 2; Table S3). These genes mainly fall into clusters of orthologous groups (COGs) of protein categories S (function unknown), M (cell wall/membrane/envelope biogenesis), D (cell cycle control, cell division, and chromosome partitioning), U (intracellular trafficking, secretion, and vesicular transport), and O (posttranslational modification, protein turnover, and chaperones). Based on these findings, it can be inferred that iYH543 encompasses the majority of essential metabolic enzymes and transporters for *S. pyogenes* serotype M1.

### Sole carbon source utilization profiles in *S. pyogenes* serotype M1

**Fig 3:**
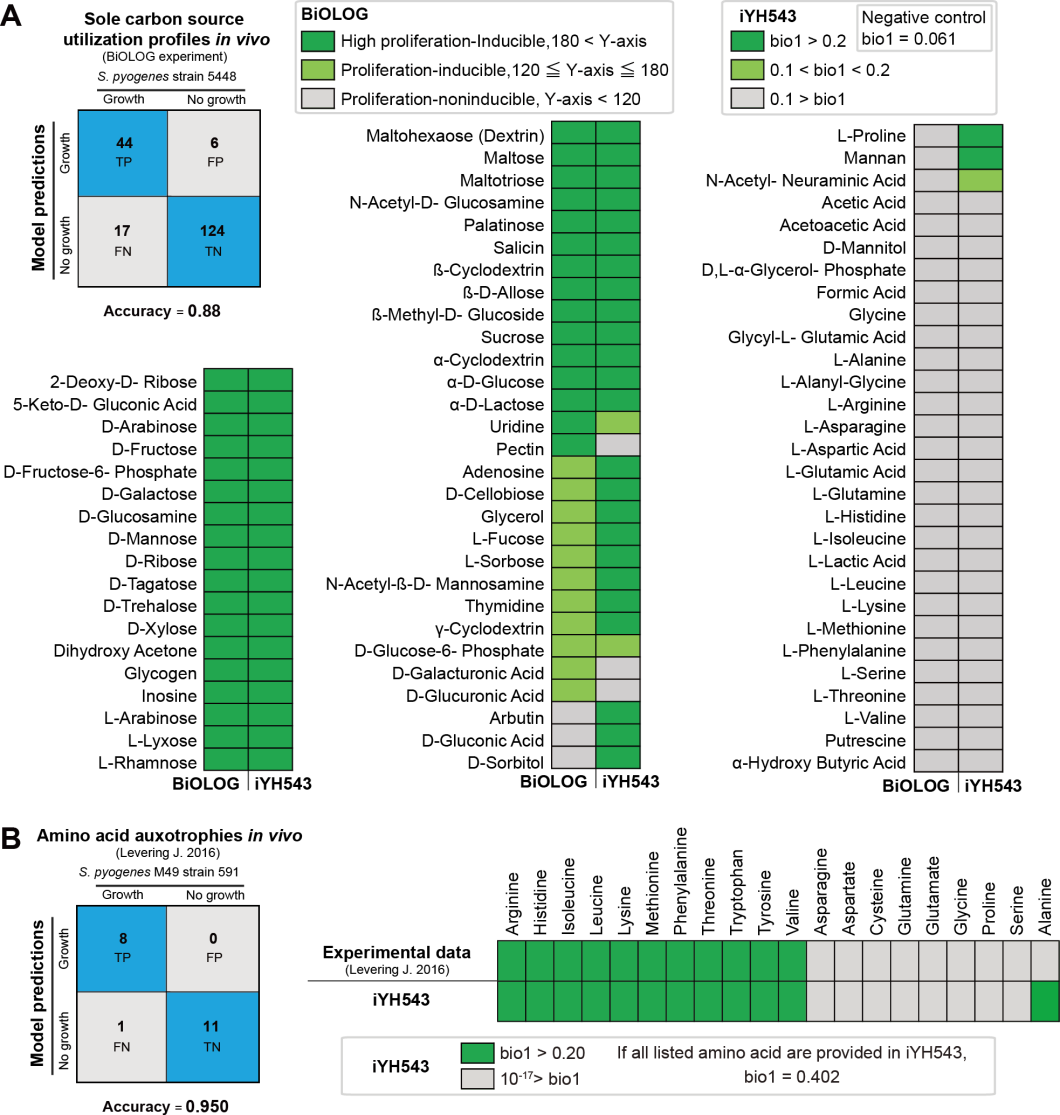
Sole carbon source utilization profiles and amino acid auxotrophies in iYH543 versus experimental data. (**A**) Biolog validation. We tested 190 carbon sources to investigate the sole carbon source utilization profiles of *S. pyogenes* M1 serotype (strain 5448). The results of the Biolog experiment are visualized in Fig. S1. The details of the medium for the simulation in iYH543 are shown in Table S6 (CDM1). The results of the simulation in iYH543 are in Table S4 (Carbon_sources). TP contains the carbon sources that allow *S. pyogenes* growth in both Biolog plates and DataS2_iYH543_CDM_1.json. TN contains (i) the carbon sources that do not allow the growth of *S. pyogenes* in both Biolog plates and DataS2_iYH543_CDM_1.json and (ii) the carbon sources that do not allow the growth of *S. pyogenes* in Biolog plates and are not exchanged in iYH543. FP contains the carbon sources that allow *S. pyogenes* growth in DataS2_iYH543_CDM_1.json, but they do not allow *S. pyogenes* growth in Biolog plates. FN contains the carbon sources that allow *S. pyogenes* growth in Biolog plates but do not allow growth in DataS2_iYH543_CDM_1.json due to the absence of the exchange reactions in BiGG or VMH. (**B**) Amino acid auxotrophies in living bacterial cells. Levering et al. reported the amino acid auxotrophies of *S. pyogenes* serotype M49 (strain 591) in living bacterial cells. We compared amino acid auxotrophies in iYH543 versus experimental data using DataS3_iYH543_CDM_2.json. The details of the medium for these simulations are shown in Table S6 (CDM2). The json formats (iYH543_CDM_1.json and DataS3_iYH543_CDM_2.json) can be downloaded at our lab website (http://www.dent.osaka-u.ac.jp/wp-content/uploads/2024/04/Code_Model_Files_Hirose_et_al.zip). The results of the simulations are in Table S4 (Amino_acid_Auxotrophy). CDM2 components and amino acid auxotrophy (11). Accuracy: (TP + TN)/all genes.

We utilized the Biolog microarray system (21) to explore the growth capabilities of *S. pyogenes* strain 5448 in chemically defined medium 1 (CDM1) media (4) with 190 different carbon sources. Our analysis identified 60 carbon sources that supported growth (Fig. 3A; Fig. S1; Table S4). Notably, CDM1 without carbohydrate sources could not sustain growth, although bacterial viability (measured as colony-forming units, CFU) remained stable for at least 8 hours. Out of the 60 carbon sources, 44 were associated with exchange reactions in the BiGG database. Hence, we incorporated the corresponding into iYH543 to enable the utilization of these 44 carbon sources for growth, relying on reactions defined in the BiGG models (Table S1). For model simulations, we employed the CDM1 medium described in Table S6 (or see DataS2_iYH543_CDM_1.json at http://www.dent.osaka-u.ac.jp/wp-content/uploads/2024/03/Code_Model_Files_Hirose_et_al.zip). The accuracy of iYH543 in predicting sole carbon source utilization profiles was 88% (168/190 carbon sources, true positive: 44, true negative: 124). Notably, the false positive group included the amino acids L-proline and L-serine. By calculating shadow prices in FBA with CDM1 medium, we determined that iYH543 utilized these amino acids for the production of cell wall-associated biomass components (reactants in biomass reaction listed in bio1 formula). Consequently, under CDM1 conditions, living bacterial cells may be unable to shift L-proline or L-serine from sources for cell wall synthesis to alternative carbon sources.

### Validation of amino acid auxotrophies in iYH543

To assess the amino acid auxotrophies in iYH543, we used published experimental data from serotype M49 *S. pyogenes* strain 591 (11) (medium specified in Table S6, or see DataS3_iYH543_CDM_2.json at http://www.dent.osaka-u.ac.jp/wp-content/uploads/2024/03/Code_Model_Files_Hirose_et_al.zip). We compared these data with simulations of iYH543 growth in CDM2, where each of the 20 amino acids was omitted individually (Fig. 3B; Table S4). Only alanine exhibited a discrepancy (false negative) between the experimental data and iYH543. In iYH543, alanine serves as a biomass component and is required for the production of peptidoglycan polymer (PGP_c). In a nutrient-rich medium, iYH543 can obtain L-alanine from peptides or via exchange reactions for L-alanine (EX_ala__L_e). However, it seems that in CDM2 (peptide-free CDM) without L-alanine, *S. pyogenes* serotype M1 is unable to synthesize alanine from other metabolites.

**Fig 4:**
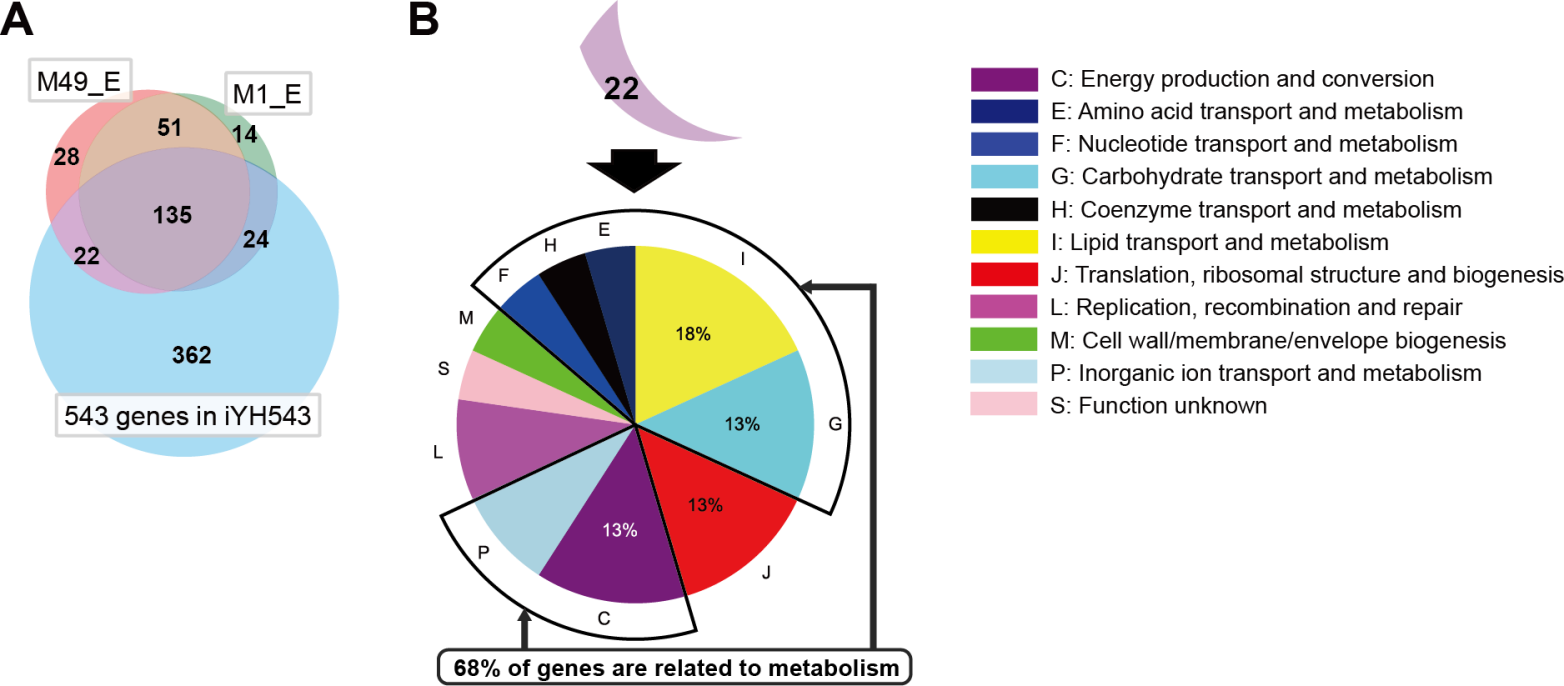
The similarity and difference of essential genes between serotype M49 NZ131 and serotype M1 SF370. (**A**) Overlapping genes among M49 essential genes, M1 essential genes, and 543 genes in iYH543. M49_E, 236 essential genes of serotype M49 NZ131. M1_E, 224 essential genes of serotype M1 SF370. Transposon mutagenesis-based gene essentiality is referenced (18). (**B**) Details of 22 essential genes of M49 NZ131 in iYH543, which are not essential in M1 SF370. COGs are colored for each function. The details of genes are listed in Table S5.

*S. pyogenes* serotype M1 and M49 share high sequence similarity, with 99.1% nucleotide identity covering 94% of the genome (serotype M49 NZ131 vs serotype M1 SF370, nBlast), which is the key reason that we used amino acid auxotrophies of serotype M49 for the curation of the serotype M1 GEM. On the other hand, Le Breton et al. reported differences in essential genes between serotype M1 and M49 when cultured in the same conditions (18). Comparing serotypes M1 and M49 among 543 genes in iYH543, 22 essential genes of serotype M49 are not essential for serotype M1. Of these 22 genes, 68% are related to metabolism (Fig. 4; Table S5). Thus, when performing curation based on the gene essentiality for GEM, it is necessary to use a serotype-specific draft. Therefore, in iYH543, we reflect amino acid auxotrophies of serotype M49 and the gene essentiality of serotype M1.

### Curation using discrepancies with experimental data

We improved the AGORA2-derived draft GEM by incorporating reactions and modifying biomass components based on *S. pyogenes* growth in CDM1 (strain 5448) (4) and CDM2 (strain 591) (11). Table S1 provides full details of this curation process. We added reactions sourced from the BiGG or VMH databases and applied curation based on available physiological data, even in cases where the corresponding GPR was unknown. GPR rules were determined based on the *S. pyogenes* pathways suggested by BioCyc or EC number information obtained from EggNOG.

**Fig 5:**
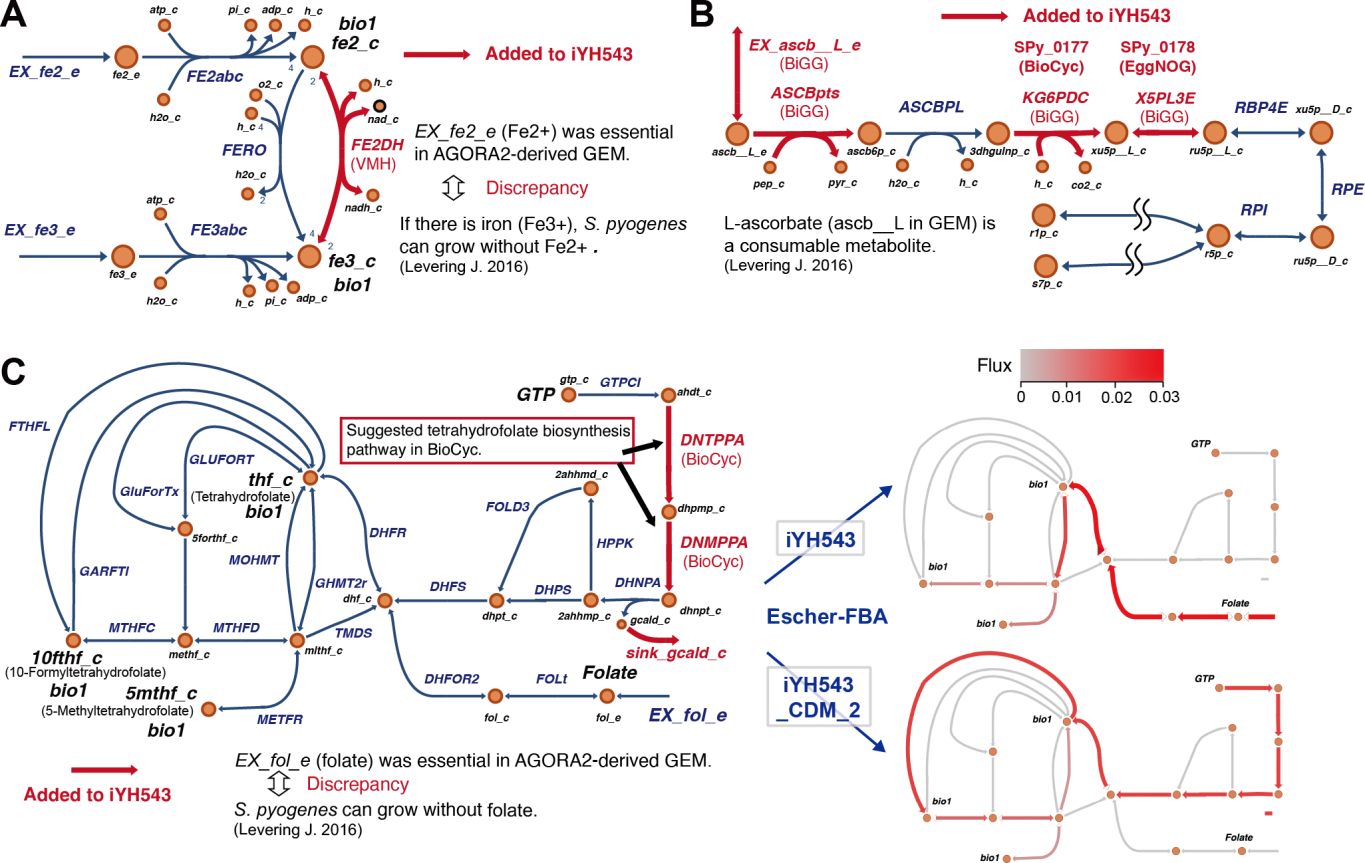
Curation of iYH543 using discrepancies between experimental data and the AGORA2-derived draft GEM. (**A**) Rationale for adding the FE2DH reaction. Fe^2+^ is a component of biomass in AGORA2-derived draft GEM; however, *S. pyogenes* can grow without Fe^2+^ in CDM2 (11). FE2DH reaction was referred from the VMH database (14). (**B**) Curated pathways for L-ascorbate degradation. Since L-ascorbate (ascb__L in GEM) is a consumable metabolite in CDM2 (11), L-ascorbate utilization reactions were added to the GEM referred from the BioCyc database (15). (**C**) Curated tetrahydrofolate biosynthesis pathway and its flux in iYH543 with a nutrient-rich media or CDM2. AGORA2-derived draft GEM requires folate (fol in GEM) in the medium, while folate is not contained in CDM2. Therefore, dihydroneopterin triphosphate pyrophosphatase (DNTPPA), dihydroneopterin monophosphate dephosphorylase (DNMPPA), and sink_gcald_c reactions were added to the GEM referred from the BioCyc database. Escher-FBA (22) was used to confirm whether the curated pathways actually worked in iYH543. Added reactions were referred from databases, including VMH, BiGG, EggNOG, and BioCyc. CDM2 components that make *S. pyogenes* growth (11). bio1, a component of biomass (a reactant in the formula for biomass, bio1 reaction); flux, estimated flux in each model.

For instance, CDM2 exclusively contains Fe^2+^ as an iron source, while the AGORA2-derived GEM lacks an enzyme to convert Fe^2+^ (fe2 in GEM) to Fe^3+^ (fe3 in GEM). However, both fe2 and fe3 are listed as biomass components in the AGORA2-derived draft GEM. To address this discrepancy, we introduced the Fe^2+^ NAD oxidoreductase (FE2DH) reaction (Fig. 5A).

In *S. pyogenes* cultured in CDM2, L-ascorbate (ascb__L in GEM) is consumed (11). However, the AGORA2-derived draft GEM did not include transport and metabolic reactions for L-ascorbate. Consequently, we added the necessary reactions to enable iYH543 to utilize L-ascorbate (Fig. 5B).

The AGORA2-derived draft GEM incorrectly predicted the requirement of folate (fol in GEM) for growth in CDM2. To rectify this, we added the DNTPPA, DNMPPA, and the sink reaction for glycolaldehyde (sink_gcald_c) reactions to iYH543 (Fig. 5C). These reactions were identified by the BioCyc database as a part of the tetrahydrofolate biosynthesis pathway in *S. pyogenes*. Escher-FBA (22) was employed to confirm whether iYH543 and iYH543_CDM_2 (see json formats at http://www.dent.osaka-u.ac.jp/wp-content/uploads/2024/03/Code_Model_Files_Hirose_et_al.zip) utilized folate to generate biomass components (thf, 5,6,7,8-tetrahydrofolate; 10fthf, 10-formyltetrahydrofolate; 5mthf, 5-methyltetrahydrofolate). The calculated flux indicated that iYH543 utilized folate for biomass component generation, while iYH543_CDM_2 relied on the curated pathways due to the absence of folate in the growth medium.

In the AGORA2-derived draft GEM, cysteinylglycine (cgly in GEM) was used to generate glutathione (gthrd in GEM) as a biomass component. However, neither CDM1 nor CDM2 required cysteinylglycine (cgly in GEM) for growth, suggesting the presence of an alternative pathway for glutathione synthesis in *S. pyogenes*. In *Streptococcus agalactiae,* glutathione synthesis occurs through a protein homologous to *Escherichia coli* d-Ala, d-Ala ligase (23). We found structural similarity between *S. pyogenes* d-Ala, d-Ala ligase (encoded by SPy_1421) and *E. coli* d-Ala, d-Ala ligase (DELTA-BLAST: query cover 99%, *E*-value 1.00*E*−126, Per. Ident 29.3%; Fig. S2). Consequently, we introduced the associated glutathione synthetase (GTHS) reaction to the model. Additionally, in *Saccharomyces cerevisiae*, form γ-glutamylcysteine (γ-L-glutamyl-L-cysteine, glucys in GEM) is formed by the condensation of γ-glutamyl phosphate (L-glutamate 5-phosphate, glu5p in GEM) and cysteine without the involvement of enzymes (24). Although the yeast *S. cerevisiae* is distant in evolutionary relationship to *S. pyogenes*, γ-glutamylcysteine synthesis from γ-glutamyl phosphate and cysteine also occurs in the closely related bacterium *S. agalactiae* (23). Therefore, we added the γ-glutamylcysteine synthesis (GLUCYSS) reaction to iYH543.

### Curation based on the gene essentiality

**Fig 6:**
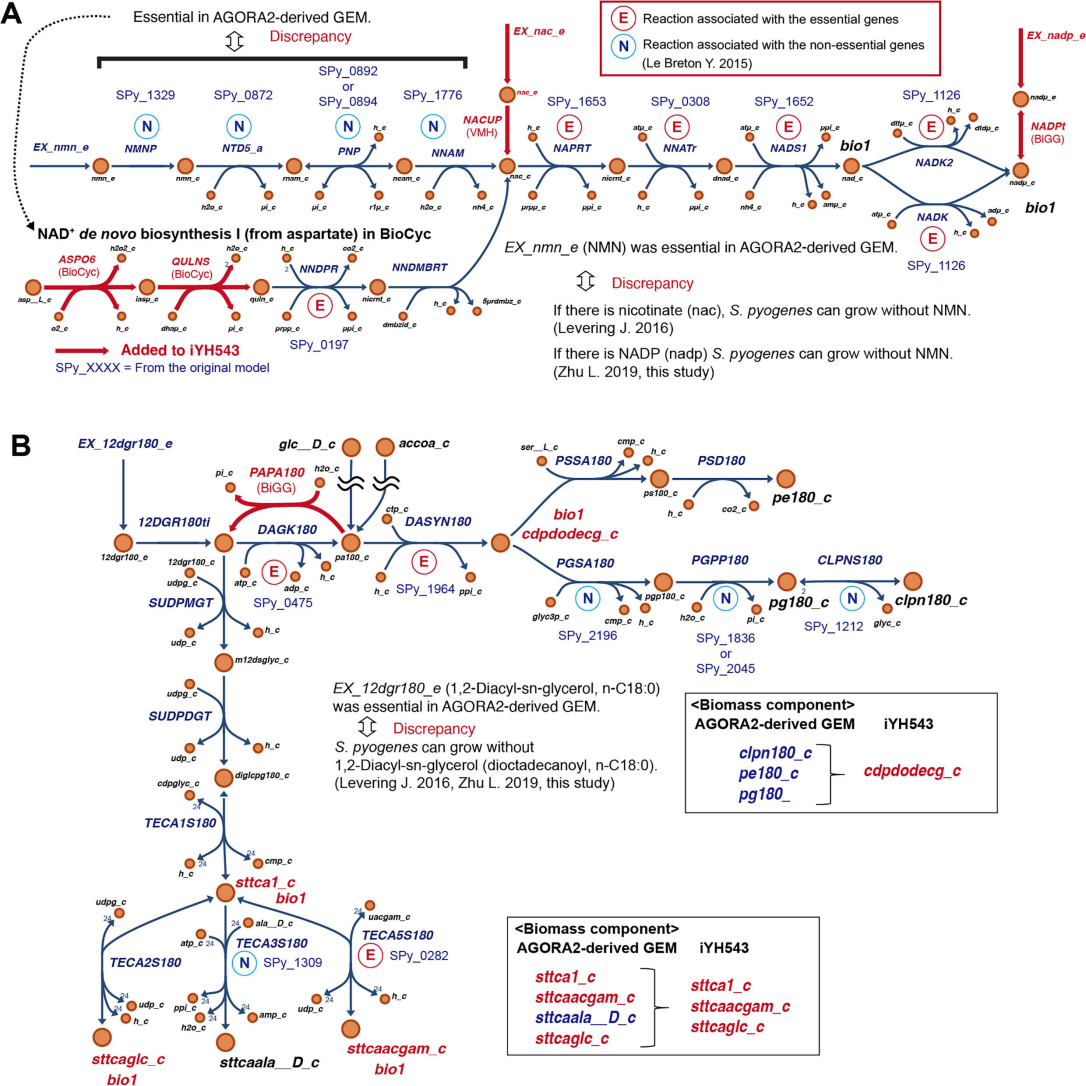
Curation of iYH543 based on transposon mutagenesis-based gene essentiality. (**A**) The curation of NAD^+^ *de novo* biosynthesis pathway. Visualization of transposon mutagenesis-based gene essentiality (18) allowed us to identify that there is no NAD^+^ *de novo* biosynthesis pathway present in the AGORA2-derived draft GEM. Therefore, by tracing NAD^+^ *de novo* biosynthesis I (from aspartate) of *S. pyogenes* in BioCyc (15), ASPO6 and QULNS reactions were added to the model. (**B**) Example for the modification of biomass components based on the transposon mutagenesis-based gene essentiality. Visualization of transposon mutagenesis-based gene essentiality (18) allowed us to distinguish between essential and non-essential biomass components of living bacteria. Biomass components associated with non-essential genes (clpn180_c, pe180_c, pg180_c, and sttcaala__D_c) are deleted from the biomass reaction (bio1 formula), and the coefficients are assigned to the precursor (cdpdodecg_c, sttca1_c) in the biomass reaction. Added reactions were referred from databases, including VMH, BiGG, and BioCyc. All reactions, metabolites, and GPR rules in iYH543 are detailed in Table S2 and can be found in the json format at our lab website (http://www.dent.osaka-u.ac.jp/wp-content/uploads/2024/04/Code_Model_Files_Hirose_et_al.zip). bio1, a component of biomass (a reactant in formula for biomass, bio1 reaction). CDM2, components that make *S. pyogenes* growth (11); CDM1, components that make *S. pyogenes* growth (4); NMN, nicotinamide mononucleotide; NADP, nicotinamide adenine dinucleotide phosphate.

The draft GEM underwent cross-referencing with a transposon mutagenesis-based gene essentiality screen conducted in *S. pyogenes* M1 (strain 5448) for growth in a “rich” THY medium (18). Discrepancies between the experimental data and reactions in the GEM were addressed through modifications. For example, the transport and metabolism pathway of nicotinate (nac in GEM) and nicotinamide adenine dinucleotide phosphate (nadp in GEM) were added to account for discrepancies with published experimental data (4, 11). Upon examining the gene essentiality (18) in the pathway depicted in Fig. 6A, a key point was identified between the nicotinamidase (NNAM) and nicotinate phosphoribosyltransferase (NAPRT) reactions, where the gene essentiality shifted from non-essential to essential. This observation suggests the existence of an alternative nicotinate synthesis pathway in *S. pyogenes*. To address this, we incorporated the L-aspartate oxidase (ASPO6) and quinolinate synthase (QULNS) reactions into the model by referencing NAD^+^ *de novo* biosynthesis I (from aspartate) in *S. pyogenes* strain SF370 from BioCyc.

The mapping of essential genes and non-essential genes allowed us to make adjustments to the biomass composition. In the AGORA2-derived draft GEM, certain genes involved in the production of cardiolipin (n-C18:0) (clpn180 in GEM) and phosphatidylglycerol (n-C18:0) (pg180 in GEM), namely, phosphatidylglycerol phosphate phosphatase (n-C18:0) (PGPP180) and stearoyl-cardiolipin synthase (CLPNS180), were marked as essential. However, in the genome-scale mutant screen, they were found to be non-essential (18). Conversely, the genes responsible for diacylglycerol kinase (n-C18:0) (DAGK180) and CDP-diacylglycerol synthetase (n-C18:0) (DASYN180), which contribute to the production of CDP-1,2-dioctadecanoylglycerol (cdpdodecg in GEM), were essential in the mutant screen (Fig. 6B).

These findings indicate that *S. pyogenes* can survive even if one of the biomass components related to CDP-1,2-dioctadecanoylglycerol (pe180, pg180, or clpn180) is absent. However, the absence of CDP-1,2-dioctadecanoylglycerol (cdpdodecg in GEM) appears to induce cell death or cell cycle arrest according to the gene essentiality data obtained from the mutant screen (18). To maintain the stoichiometric balance in the biomass reaction (bio1) for our flux balance analysis, we consolidated clpn180, pe180, and pg180 as cdpdodecg, tripling their coefficients in the biomass reaction of iYH543 (Table S1). Similarly, the stearoyllipoteichoic acid (linked, D-alanine substituted; sttcaala__D in GEM) was removed; its coefficient was equally evenly distributed among the other three stearoyllipoteichoic acids (sttca1: linked, unsubstituted; sttcaacgam: linked, N-acetyl-D-glucosamine; and sttcaglc: linked, glucose substituted) (Fig. 6B). The biomass related to isoheptadecanoyllipoteichoic acid (i17tca1 in GEM) and CDP-1,2-diisoheptadecanoylglycerol (cdpdihpdecg in GEM), as well as anteisoheptadecanoyllipoteichoic acid (ai17tca1 in GEM) and CDP-1,2-dianteisoheptadecanoylglycerol (cdpdaihpdecg in GEM), was also re-assigned by using the same approach (Fig. S3 and S4). The json formats for pathway maps (DataS4_i17tca1_cdpdihpdecg.json and DataS5_ai17tca1_cdpdaihpdecg.json) can be downloaded at our lab website (http://www.dent.osaka-u.ac.jp/wp-content/uploads/2024/03/Code_Model_Files_Hirose_et_al.zip).

In the draft GEM, the 1,2-diacyl-sn-glycerol (dioctadecanoyl, n-C18:0) exchange (EX_12dgr180_e) reaction was considered essential. However, live *S. pyogenes* can grow without 1,2-diacyl-sn-glycerol (dioctadecanoyl, n-C18:0) (12dgr180 in GEM) in both CDM1 (4) with glucose and CDM2 (11). In the AGORA2-derived draft GEM, the presence of 12dgr180_e was required to produce stearoyllipoteichoic acids (linked, unsubstituted; sttca1 in GEM)-related biomass components. To address this, we added the phosphatidate phosphatase (n-C18:0, PAPA180) reaction to the model without a GPR association. This modification allowed iYH543 to generate sttca1-related biomass components from D-glucose (glc__D in GEM) or acetyl-CoA (accoa in GEM) (Fig. 6B).

### Drastically modified metabolic pathways from the AGORA2-derived draft GEM

Although the meso-2,6-diaminoheptanedioate exchange (Ex_26dap_M_e) reaction is considered essential in the AGORA2-derived draft GEM, *S. pyogenes* can actually grow in CDM2 without meso-2,6-diaminoheptanedioate (26dap_M in GEM) CDM2 (11). However, the genes associated with the peptidoglycan synthesis pathway from 26dap_M_c to PGP_c (peptidoglycan polymer) are listed as essential genes (18), and there are no alternative pathways in iYH543 to produce PCP_c. To address this, we added all the reactions of the meso-2,6-diaminoheptanedioate (26dap_M in GEM) synthesis pathway from L-aspartate (asp__L in GEM), based on the pathway found in the iYS854 model, a *Staphylococcus aureus* GEM (25) (Fig. S5A). It is worth noting that *S. aureus* and *S. pyogenes* are both Firmicutes and cause similar soft tissue infections (26, 27).

The lipid synthesis pathway underwent significant modifications (Fig. S5B). In *S. pyogenes*, exogenous biotin is necessary for growth (28). However, the AGORA2-derived draft GEM erroneously predicted that exogenous biotin is not required. Moreover, the pathway for lipid synthesis from biotin includes several essential genes in *S. pyogenes* serotype M1 (18). To rectify this inconsistency, we removed the acetyl coenzyme A carboxylase (ACCOAC) reaction and introduced the biotin--[biotin carboxyl-carrier protein] ligase (BBCCPLi) reaction, as well as the demand reactions for Apoc-Lysine (DM_apoC_Lys_c) and apoprotein [acyl carrier protein] (DM_apoACP_c). Additionally, we adjusted the GPR for the biotin transport via ABC system (BTNabc) reaction, as studies have shown that biotin transport in *S. pyogenes* is facilitated by *bioY* (SPy_0207) or cobalt transporter homologs encoded by *cbiOQ* (SPy_1787, SPy_1788) (29, 30).

The fatty acid biosynthesis pathway was enhanced by incorporating the SPy_1746 (*fabZ*), SPy_1748 (*fabF*), SPy_1749 (*fabG*), SPy_1751 (*fabK*), and SPy_1758 (*fabM* or *phaB*) reactions (Fig. S6) based on pathways from the BioCyc database (15) and findings in *Streptococcus pneumoniae* (31). The AGORA2-derived draft GEM lacked unsaturated fatty acids in its biomass components, despite C16:1 and C18:1 monounsaturated fatty acids being constituents of the *S. pyogenes* cell membrane (32, 33). Although the draft GEM did not contain synthesis pathways for palmitate (C16:0) or unsaturated fatty acids, it did contain glucosyl diacylglycerol (Glc-DAG) synthesis pathways for CDP-1,2-dihexadecanoylglycerol (16:0/16:0) (cdpdodec11eg_c) and CDP-1,2-dioctadec-11-enoylglycerol (18:1/18:1) (cdpdhdecg_c). Hence, cdpdodec11eg_c and cdpdhdecg_c were added to the biomass reaction (bio1 formula). Notably, Glc-DAG, which can include C16:0 and C18:1 and is the predominant glycolipid among *S. pneumoniae*, *S. pyogenes,* and *S. agalactiae* (33, 34), exhibits specific recognition by invariant T cell receptors of natural killer T cells (34).

### Hypotheses for explaining the false positives and false negatives in iYH543

The curation of iYH543 incorporated available physiological data, even in cases with unknown GPRs. However, there were remaining genes in iYH543 that showed discrepancies in predicting gene essentiality, classified as FP or FN. These discrepancies led us to formulate several hypotheses (detailed in Table S3).

For example, SPy_1746 (*fabZ*) and SPy_1751 (*fabK*), involved in the fatty acid biosynthesis pathway (Fig. S6, or see DataS6_fatty_acid_bioshynthesis.json at http://www.dent.osaka-u.ac.jp/wp-content/uploads/2024/03/Code_Model_Files_Hirose_et_al.zip), were classified as FN, meaning they were deemed essential in iYH543 but non-essential in live bacterial cells cultured in the THY medium (18). This is likely because *S. pyogenes* is able to utilize exogenous fatty acids from rich media or the host via the fatty acid kinase system, in lieu of the *de novo* production of fatty acids via FabZ and FabK (35).

FPs refer to genes reported as non-essential by iYH543 but found to be essential in living bacterial cells cultured in the THY medium. One such FP is SPy_1315 (ABC transporter permease subunit, *glnP*), which serves as a GPR for L-glutamine transport via the ABC system (GLNabc) reaction in iYH543. Since L-glutamine can be produced from other components in iYH543 (Fig. S7, or see DataS7_core_metabolism.json at http://www.dent.osaka-u.ac.jp/wp-content/uploads/2024/03/Code_Model_Files_Hirose_et_al.zip), the GLNabc reaction (with Spy_1315 in its GPR) is considered non-essential in iYH543. However, a DELTA-BLAST search reveals that *S. pyogenes* GlnP (encoded by SPy_1315) shares high similarity with a part of an ABC transporter involved in lysine, arginine, and ornithine transport in *Streptococcus infantarius* subsp. (accession: EDT46715.1) (query cover 100%, *E*-value 1.00*E*−126, Per. ident 72.7%). The L-lysine transporter (LYSt2r) reaction is essential and lacks a GPR in iYH543, suggesting that SPy_1315 may be responsible for L-lysine transport. In summary, iYH543 and the provided json maps in this study serve as valuable resources for investigating the unknown metabolic mechanisms of *S. pyogenes*.

### Hypothesis and knowledge obtained by substituting actual measurement values for iYH543

We previously reported that the hemolytic activities of *S. pyogenes* M1 serotype are dramatically altered by different carbon sources in CDM1 (36). *S. pyogenes* has two hemolysins, streptolysin O (SLO) and streptolysin S (SLS), which contribute to the formation of necrotizing skin lesions (37, 38). In our previous study, glucose, maltose, and dextrin caused hemolysis in SLO-dependent, SLS-dependent, and both SLO- and SLS-dependent manners, respectively (36). However, the mechanism behind this phenomenon remained unclear. Especially, it was difficult to explain why dextrin could upregulate both hemolysins.

**Fig 7:**
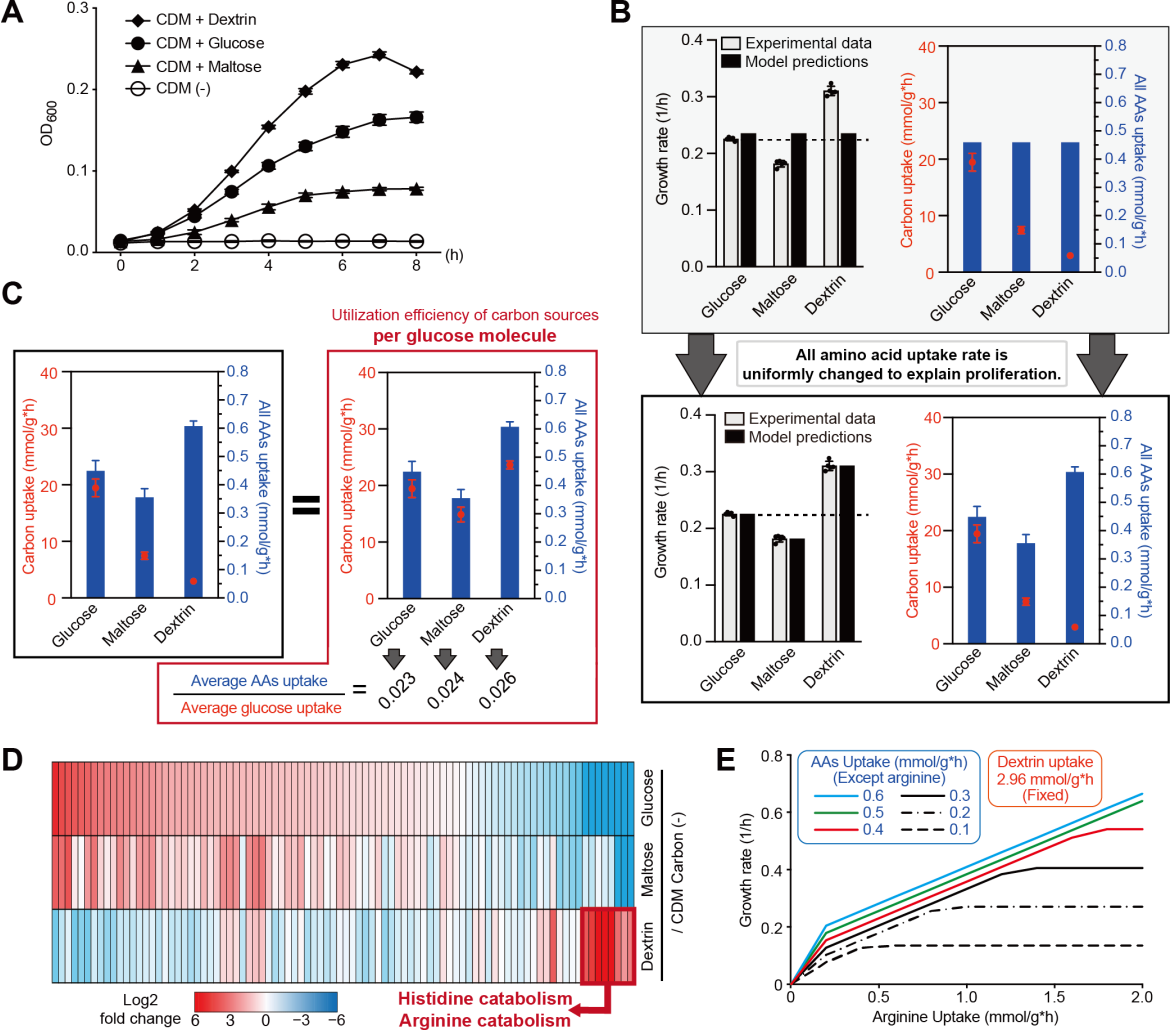
Utilization efficiency of amino acid is changed by the difference of supplemented carbon sources in CDM1. (**A**) Growth curves in CDM1 supplemented with indicated carbon sources. (**B**) Calculated differences in the efficiency of AA uptake (mmol/g*h) in CDM1 glucose (+), maltose (+), or dextrin (+) conditions. Assuming that the efficiency of AA uptake is constant regardless of carbon sources, iYH543 predicts the same growth rates in all CDM1 glucose (+), maltose (+), or dextrin (+) conditions (above, gray). The efficiency of AA uptake is adjusted according to the actual values of growth rate (1/h) and carbon uptake (mmol/g*h) (bottom). (**C**) Calculated differences in the efficiency of AA uptake per glucose molecule uptake in CDM1 glucose (+), maltose (+), or dextrin (+) conditions. The simulation suggested that maltose utilization tends to show inefficient amino acid uptake as compared to glucose utilization. On the other hand, the simulation suggested that glucose utilization tends to show efficient amino acid uptake as compared to glucose utilization. (**D**) The expression of selected genes in iYH543, associated with amino acid transport and metabolism. Bacterial RNA samples from CDM1 glucose (+), maltose (+), or dextrin (+) conditions are compared to those from CDM1 carbon (−) conditions. These data are acquired from a previous study (36). The color scale indicates enrichment (red) and depletion (blue). Genes are arranged in descending order of log2 fold change values in CDM1 glucose (+) condition. The detail of the expression levels is shown in Table S7. (**E**) The growth rate in proportion to the arginine uptake in iYH543 with CDM dextrin (+) condition. The actual consumption rates of dextrin 2.96 mmol g^−1^ h^−1^ are used as the fixed value. AA uptake, lower bounds of exchange reactions of other all AAs; arginine uptake, flux of the exchange reaction of arginine.

To investigate the metabolism involved in bacterial hemolytic activities, we used iYH543. First, we measured the growth parameters in CDM1 glucose (+), maltose (+), or dextrin (+) conditions (Fig. 7A). The specific growth rates on glucose, maltose, and dextrin during the exponential phase were 0.229, 0.182, and 0.311 h⁻¹, respectively. The specific consumption rates of glucose, maltose, and dextrin were 19.40, 7.44, and 2.96 mmol g^−1^ h^−1^, respectively. However, when substituting the measured specific consumption rate of glucose, maltose, and dextrin for iYH543, the predicted growth rates by FBA did not match the actual growth rate (Fig. 7B). When simulating the change of uptake efficiency (lower bounds) of medium components in iYH543, we found that the uptake efficiency of amino acids is directly related to the growth rate in FBA. In iYH543, the lower bounds for all amino acids (AAs) in the medium are set to one-tenth that of carbon sources (Table S6). Therefore, we uniformly modified the lower bounds for the uptake reaction for all AAs in iYH543 to match the actual growth and consumption rates (Fig. 7B). Maltose is a disaccharide of D-glucose. Dextrin is represented in iYH543 by eight linked polymers of D-glucose, because dextrin (starch) is consumed as γ-cyclodextrin (the eight glucose subunits are linked end-to-end via α-1,4 linkages) in the model. When describing the utilization efficiency of all AAs and carbon sources per glucose molecule in each condition (Fig. 7C), iYH543 predicts that not only the difference in the uptake rate of carbon sources but also that of amino acids affects the growth rates.

To compare the gene expression associated with amino acid utilization in each condition, transcriptome data were extracted from our previous data set (36) (Fig. 7D; Table S7). Bacterial RNA samples from the CDM1 glucose (+), maltose (+), or dextrin (+) conditions were compared to those from the CDM1 carbon (−) condition. In the CDM1 maltose (+) condition, the expression levels of AA metabolic genes seemed to decrease overall compared to the CDM1 glucose (+) condition. In comparisons of the CDM1 dextrin (+) and glucose (+) conditions, gene expression profiles are significantly altered, with histidine and arginine utilization pathways being dramatically upregulated in the CDM1 dextrin (+) condition. As a result of FBA by using iYH543 in the CDM dextrin (+) condition, the growth rate is increased in proportion to the arginine uptake (flux of the exchange reaction of arginine) despite the low uptake (lower bounds of exchange reactions) of all other AAs (Fig. 7E). On the other hand, increasing histidine uptake did not affect the growth rate in FBA by using iYH543 in the CDM dextrin (+) condition. This suggests that enhanced arginine catabolism is associated with the high proliferation ability induced by dextrin. Additionally, we previously indicated that the upregulation of arginine catabolism is linked to the increased expression of both SLS and SLO-encoding genes (6). Together, the knowledge obtained by substituting actual measurement values for iYH543 helps us to gain unknown knowledge that connects metabolism and virulence.

## DISCUSSION

The fully curated GEM, iYH543, is the first GEM for *S. pyogenes* serotype M1 and achieved 92.6% accuracy in predicting gene essentiality. This accuracy is a significant advance upon the previously reported *S. pyogenes* serotype M49 GEM (76.6%), making iYH543 especially valuable for the study of serotype M1 strains, which are typically more pathogenic and clinically relevant. The GEM construction strategy employed here also serves as a template for curating highly accurate and consistent GEMs. The curation process provided unique insights that would have been difficult to obtain through any other means, establishing connections between previous knowledge and new discoveries in *S. pyogenes* metabolism.

In this study, we described the pathway map for cell wall synthesis and nucleotide metabolism (Fig. S8 and S9, respectively) (see DataS8_cell_wall.json and DataS9_nucleotide.json at http://www.dent.osaka-u.ac.jp/wp-content/uploads/2024/03/Code_Model_Files_Hirose_et_al.zip). The predicted pathways are based on actual experimental data such as the essential gene information or the nutrient uptake information, making iYH543 more accurate than pathway maps based on genome information alone (39).

Interestingly, among the eight genes present in iYH543, but not M49 strains, is an operon for maltose or dextrin utilization (*malCDX* or *amyAB*) (Fig. S10B). Our previous research showed an *S. pyogenes* M1 strain upregulated both *nga-ifs-slo* operons and an operon for maltose or dextrin utilization at the same time. These co-upregulated gene sets (called as MalR2 iModulon) contributed to the upregulation of *nga-ifs-slo* operons in the CDM1 maltose (+) or dextrin (+) conditions (Fig. S10C) (36). The *nga-ifs-slo* operon encodes an NAD glycohydrolase (NADase), immunity factor, and pore-forming cytolytic toxin SLO that contribute to enhanced virulence of *S. pyogenes* (40, 41).

To utilize dextrin, *S. pyogenes* must break it down into glucose molecules. Even though dextrin utilization requires more enzyme effort than glucose utilization, our results showed that the actual growth efficiency was higher in the CDM1 dextrin (+) condition as compared to the CDM1 glucose (+) condition. The simulations using iYH543 suggested that differences in the utilization efficiency of amino acids may be a contributing factor. Specifically, we suggest that arginine catabolism is associated with the high proliferation ability induced by dextrin. This implies that utilizing simulations with iYH543 to interpret omics data is more effective than exploring it without guidance. Our previous study suggested that *S. pyogenes* increases the utilization of various polysaccharides, peptides, and amino acids when sensing glucose depletion in the host organism (7). This increased utilization of polysaccharides and amino acids may contribute to the expression of hemolytic toxins and the proliferation of bacteria when *S. pyogenes* is exposed to the host’s nutritional immunity *in vivo*. However, the limitation of this study is that actual amino acid consumption and enzyme costs were not reflected in the simulation. Therefore, further investigation is needed to demonstrate the additional benefits of iYH543.

Mismatches between essential genes predicted by the GEM and those observed in living bacterial cells highlighted areas of uncertainty in our current understanding of *S. pyogenes* metabolism. Further research is required to address these discrepancies. Since gene functions in both iYH543 and living *S. pyogenes* cells are inferred from database annotations, it remains unclear if these predicted functions align with actual enzymatic activities. Therefore, as in any genome-scale annotation effort, continuous efforts are required for the network reconstruction process of *S. pyogenes*. Despite these limitations, iYH543 represents a useful tool for rational drug design targeting *S. pyogenes* metabolism (10) and computational screening to investigate the interplay between inhibiting virulence factor synthesis and growth (42).

## MATERIALS AND METHODS

### Strains

The draft GEM for *S. pyogenes* serotype M1 (strain SF370) (accession no. AE004092) was obtained from AGORA2 (termed AGORA2-derived GEM) created by Heinken et al. (12). In laboratory experiments, *S. pyogenes* M1 strain 5448 (accession no. CP008776), isolated from a patient with toxic shock syndrome and necrotizing fasciitis (43), was used, because a transposon mutagenesis-based gene essentiality screen was performed using this strain (18). Orthologs between 5448 and SF370 strains were determined using bidirectional BLAST hits.

*S. pyogenes* was grown at 37°C in a screw-cap centrifuge tube (BD Biosciences, San Jose, CA, USA) filled with 10 mL Todd–Hewitt broth supplemented with 0.2% yeast extract (THY) (Hardy Diagnostics, Santa Maria, CA, USA) or CDM1 (4) in an ambient atmosphere and standing cultures. To obtain cultures for experiments, overnight cultures of *S. pyogenes* were back diluted 1:50 into fresh THY broth and grown at 37°C, with growth monitored by measuring optical density at 600 nm (OD_600_). For the experiments using CDM1, overnight culture of *S. pyogenes* in THY broth was centrifuged, resuspended to fresh CDM, and diluted 1:20 into fresh CDM1 supplemented with indicated carbon sources (4.5 g/L). Growth kinetic experiments were performed in a Tecan Infinite Pro 200 plate reader (Tecan, Mannedorf, Switzerland). Cell concentration was measured as OD_600_ every 30 min. CFUs were determined by plating diluted samples on THY agar.

### Biolog phenotyping experiments

Biolog’s Phenotype MicroArray technology (21) was used to investigate the sole carbon source utilization profiles of *S. pyogenes* strain 5448 during growth in 190 carbon sources (PM1- and PM2A-Microplates, Biolog, Hayward, CA, USA). *S. pyogenes* was grown to the mid-log phase in THY (OD_600_ = 0.3 ~ 0.5), then was centrifuged and resuspended in CDM1 (Table S6) (4). The suspension was adjusted to OD_600_ = 0.09 and mixed with Redox Dye Mix H (Biolog). One hundred microliters of suspension was added into each well of the microplate and incubated at 37°C for 48 hours. The colorimetric assay was considered as positive when the average of the *y*-axis was 1.2 times higher than that of the negative control.

### Construction of iYH543

We constructed the iYH543 model based on the AGORA2-derived draft GEM of *S. pyogenes* M1 (strain SF370). The AGORA2-derived draft GEM of *S. pyogenes* M1 serotype was generated in the AGORA2 pipeline (12, 44). Briefly, we downloaded draft genome-scale metabolic reconstructions using KBase (45). In the platform, the genome sequence of an organism is automatically annotated, and a metabolic reconstruction is assembled based on the identified metabolic functions. The imported assemblies were annotated using RAST subsystems (46). All reactions and metabolites of these draft metabolic reconstructions were translated into the VMH (14). Gaps in the draft reconstruction are automatically filled, building a metabolic reconstruction whose condition-specific models can carry flux through a defined biomass objective function. We refined the draft reconstructions using rBioNet (47) and performed quality control and quality assurance tests.

The additional curation in this study was conducted by using the protocol made by Orth et al. (19, 48). In short, the AGORA2-derived draft GEM was intensively manually curated using the information from BiGG (13), VMH (14), BioCyc (15), KEGG (16), and EggNOG-generated EC number information (17). To extract metabolite and reaction information from BiGG, all VMH database formats of the AGORA2-derived draft GEM were converted to the BiGG database format. Where possible, reactions were verified with experimental data or external data. For gap filling, pathways were visualized using Escher (20), and the gaps were filled with reactions shown in BioCyc or other BiGG models containing iB21_1397 (49), iJO1366 (50), iMM904 (51), iYO844 (52), iYS854 (25), and Recon3D (53). Escher-FBA was used to confirm the fluxes in GEMs (22). To identify homologous genes, BLASTp homology searches were done using the NCBI webserver with default settings or using DELTA-BLAST (54). Demand reactions are added to allow the accumulation of compounds in iYH543, and sink reactions are added to provide the network with metabolites. Some reactions were added based on literature information. A fully detailed rationale for each curation is listed in Table S1.

The proton/element balance in the reactions and the molecular weight of the biomass (which ideally should be 1 g/mmol) were checked by BiomassMW (55).

### Simulations using COBRApy

The reconstruction was converted to a mathematical model, using Constraint-Based Metabolic Modeling in Python (COBRApy) version 0.22.1 (56). COBRApy (solver: glpk) was also used for simulating with FBA and simulating the knockout of single genes and reactions.

All simulations on gene essentiality, amino acid auxotrophy, and carbon sources were done using FBA while optimizing for the biomass reaction. For all metabolites present in the media, the upper and lower bounds were set to −1,000 and 1,000 mmol/g/h, respectively. In FBA simulations in nutrient-rich media, we used a value of 10^−8^ as a growth/no growth cutoff.

In cases/conditions where the FBA model predicted a no-growth phenotype, while the experimental or external data suggested growth, we calculated the shadow price (57), allowing the prediction of specific metabolite accumulation or depletion preventing the growth of the FBA model (58).

### Measurement of a conversion coefficient between OD_600_ and dry cell weight

Overnight culture of *S. pyogenes* in THY broth was centrifuged, resuspended to fresh CDM1, and diluted 1:20 into 1 L of fresh CDM1 supplemented with dextrin (4.5 g/L). A screw-cap bottle was filled with the mixture and incubated in an ambient atmosphere and standing cultures. During the exponential phase, 1,000 mL of bacterial culture was filtered through a membrane filter (Cat. H050A090C, pore size: 0.5 µm) (Advantec, Tokyo, Japan) with measurement of its OD_600_. The filter with cells was dried at 60°C for 16 hours in an oven and was weighted. The conversion coefficient between OD_600_ and dry cell weight was calculated as 0.713 g L^−1^ OD_600_^−1^.

### Measurement of extracellular metabolites and specific rates

To evaluate the time-course of extracellular substrate concentration, the culture supernatants were collected every hour for 8 hours by centrifugation at 12,000 rpm for 5 min. Enzymatic assay kits were used for the measurement of D-glucose (Cat. 716251), maltose (Cat. E1268), and dextrin (Cat. 1113950) (Boehringer Mannheim, Mannheim, Germany). Specific rates for growth and substrate consumptions were determined by linear regression using the culture profile during the exponential growth phase. The calculation procedure was described elsewhere (59).

## Data Availability

The new metabolic network reconstructions for *S. pyogenes* serotype M1, iYH543, are provided in a spreadsheet format in Table S2. The json formats of iYH543, iYH543_CDM_1, iYH543_CDM_2, and AGORA2_derived_draft_GEM can be downloaded at our lab website (http://www.dent.osaka-u.ac.jp/wp-content/uploads/2024/03/Code_Model_Files_Hirose_et_al.zip). Curation notes are detailed in Table S1. The lab website also includes files detailing the development of models, simulation codes for FBA, and calculation codes for shadow price, biomass molecular weight, and growth rate in proportion to the arginine uptake.

## SUPPLEMENTAL MATERIAL

Supplemental information msystems.00736-24-s0001.docx Figures S1 to S10.

Table S1 msystems.00736-24-s0002.xlsx All information of the curation.

Table S2 msystems.00736-24-s0003.xlsx All reactions, metabolites, and GPR rules in iYH543.

Table S3 msystems.00736-24-s0004.xlsx Details of essential genes in both iYH543 and a published genome-scale mutant screen.

Table S4 msystems.00736-24-s0005.xlsx Sole carbon source utilization profiles and amino acid auxotorophies in both the experimental data and iYH543.

Table S5 msystems.00736-24-s0006.xlsx Details of genes that M49 NZ131 or M1 5448 shows essential among iYH543 genes.

Table S6 msystems.00736-24-s0007.xlsx Details of nutrient-rich medium, CDM1, and CDM2 in iYH543 and actual recipe for CDM1.

Table S7 msystems.00736-24-s0008.xlsx Expression of selected genes in iYH543, associated with the amino acid transport and metabolism.

ASM does not own the copyrights to Supplemental Material that may be linked to, or accessed through, an article. The authors have granted ASM a non-exclusive, world-wide license to publish the Supplemental Material files. Please contact the corresponding author directly for reuse.
