# Supplementary material for: A genome-scale metabolic model of a globally disseminated hyperinvasive M1 strain of Streptococcus pyogenes
Source: mSystems. 2024 Aug 19;9(9):e00736-24. doi: 10.1128/msystems.00736-24 (PMC11406949; doi:10.1128/msystems.00736-24)
Supplement: Supplemental information — Figures S1 to S10. [file msystems.00736-24-s0001.docx]

**Supplementary Information**

**A genome-scale metabolic model of a globally disseminated hyperinvasive M1 strain of *Streptococcus pyogenes***

**Authors**

Yujiro Hirose^1,2 *^, Daniel C. Zielinski^3^, Saugat Poudel^3^, Kevin Rychel^3^, Jonathon L Baker^2,4,5^, Yoshihiro Toya^6^, Masaya Yamaguchi^1,7,8,9^, Almut Heinken^10,11,12^, Ines Thiele^10,13,14^, Shigetada Kawabata^1,9^, Bernhard O. Palsson^3^, Victor Nizet^2,15 *^

**Affiliations**

^1^Department of Microbiology, Osaka University Graduate School of Dentistry, Suita, Osaka 5650871, Japan.

^2^Department of Pediatrics, University of California at San Diego School of Medicine, La Jolla, California 92093, USA

^3^Department of Bioengineering, University of California San Diego, La Jolla, CA 92093, USA.

^4^Genomic Medicine Group, J. Craig Venter Institute, La Jolla, California, USA.

^5^Department of Oral Rehabilitation & Biosciences, OHSU School of Dentistry, Portland, OR, 97202, USA

^6^Department of Bioinformatic Engineering, Graduate School of Information Science and Technology, Osaka University, Suita, Osaka, 565-0871, Japan

^7^Bioinformatics Research Unit, Graduate School of Dentistry, Osaka University, 1-8 Yamada-Oka, Suita, Osaka, 5650871, Japan.

^8^Bioinformatics Center, Research Institute for Microbial Diseases, Osaka University, 3-1 Yamada-Oka, Suita, Osaka, 5650871, Japan.

^9^Center for Infectious Diseases Education and Research, Osaka University, 2-8 Yamada-Oka, Suita, Osaka, 5650871, Japan.

^10^School of Medicine, National University of Galway, Galway, Ireland

^11^Ryan Institute, University of Galway, Galway, Ireland

^12^Inserm UMRS 1256 NGERE, University of Lorraine, Nancy, France

^13^Division of Microbiology, National University of Galway, Galway, Ireland

^14^APC Microbiome Ireland, Cork, Ireland

^15^Skaggs School of Pharmaceutical Sciences, University of California at San Diego, La Jolla, California 92093, USA

*Correspondence:

Victor Nizet, E-mail: vnizet@ucsd.edu, TEL: +18585347408

Yujiro Hirose, E-mail: hirose.yujiro.dent@osaka-u.ac.jp, TEL: +81668792897

**Table of Supplementary figures**

S1: The results of BiOLOG experiments..................................................................................................................2

S2: The evidences for adding GLUCYSS and GTHS reactions...............................................................................3

S3: The modification of i17tca1_c- and cdpdihpdecg_c- related biomass based on the gene essentiality………...4

S4: The modification of ai17tca1_c- and cdpdaihpdecg_c - related biomass based on the gene essentiality……...5

S5. Significantly modified metabolic pathway..........................................................................................................6

S6: Fatty acid biosynthesis pathway in iYH543…………………………………………………………....………7

S7: Core metabolism in iYH5433……………………………………..……………………………………........…8

S8: Cell wall synthesis pathway in iYH543…………………………………………………………………...……9

S9: Nucleotide metabolism in iYH543……………………………………………………………………………..10

S10: The similarity and difference of genomes between M49 strains and M1 SF370………………………….…...11


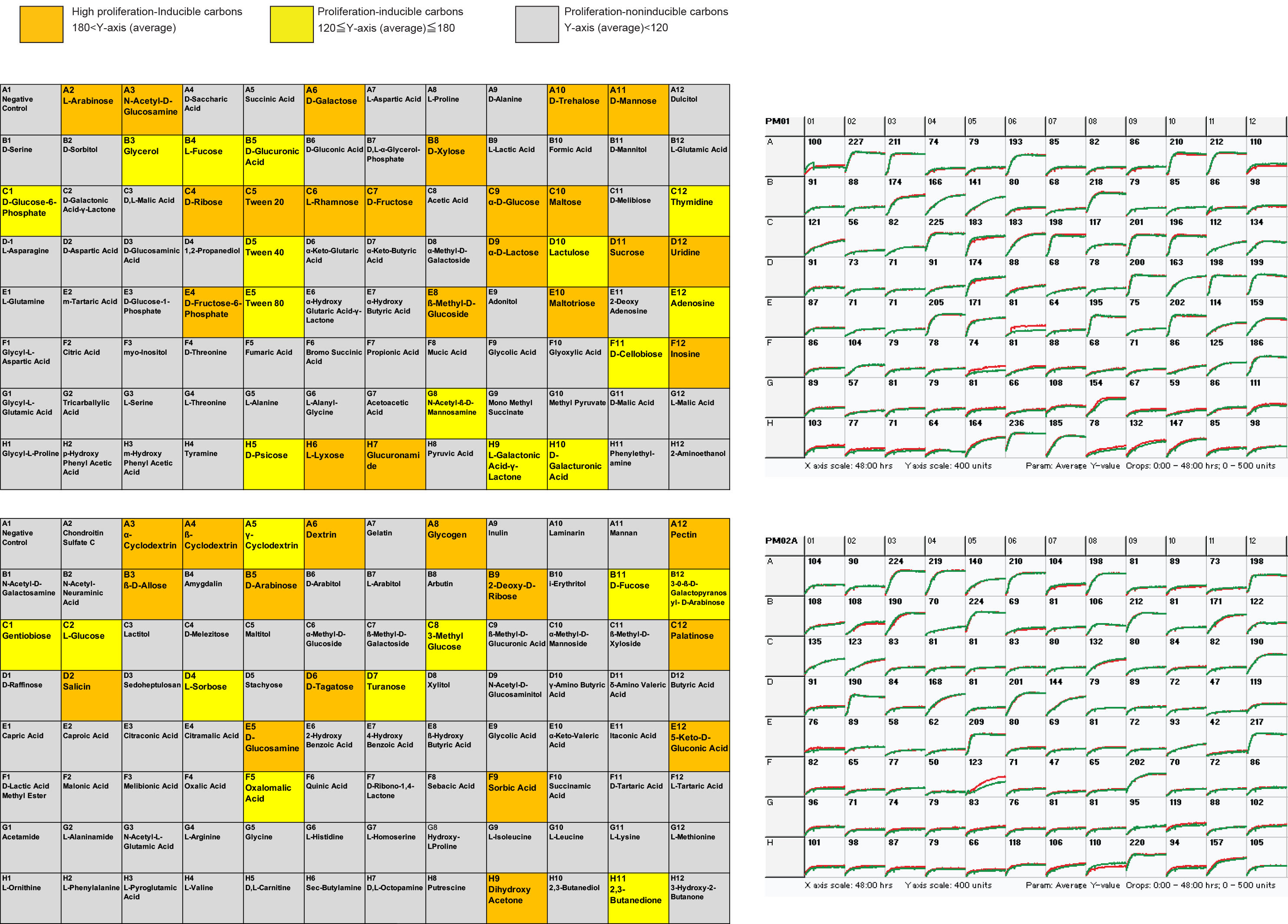
**Supplementary Figures**

**Supplementary Figure 1. The results of BiOLOG experiments, single carbon source utilization for *S. pyogenes* strain 5448.**


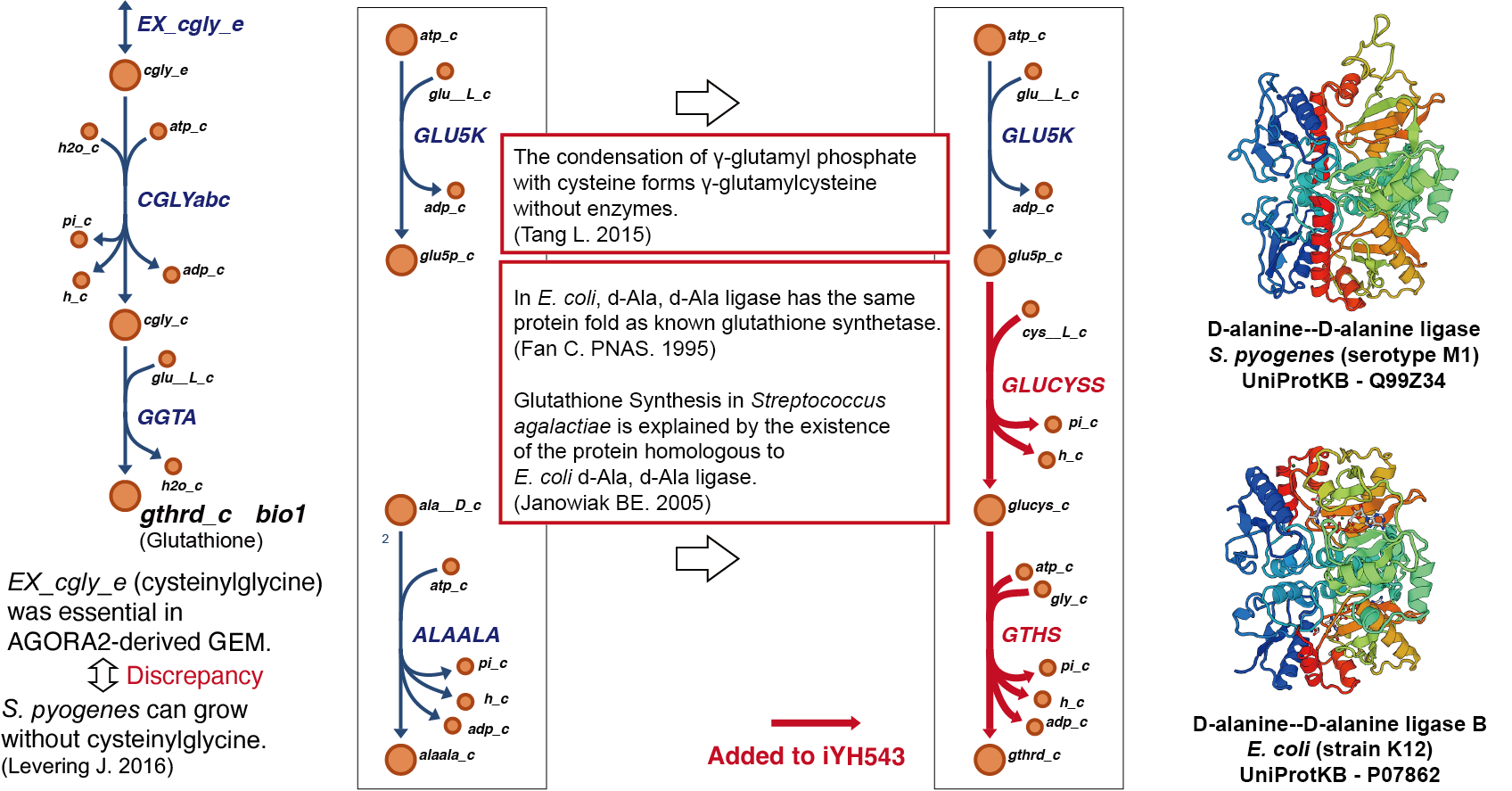
**Supplementary Figure 2. The evidences for adding GLUCYSS and GTHS reactions.** All reactions, metabolites, and gene–protein–reaction (GPR) rules in iYH543 are detailed in **Supplementary Table 1** and can be found in json format in **Supplementary Data 1**.

CDM2 components (Levering J. 2016)[1] (PMID: 26970054).

Evidence for GLUCYSS reaction (Tang L. 2015)[2] (PMID: 26377681).

Evidence for GTHS reaction (Fan C. 1995)[3] (PMID: 7862655) (Janowiak BE. 2005)[4] (PMID: 15642737).

**
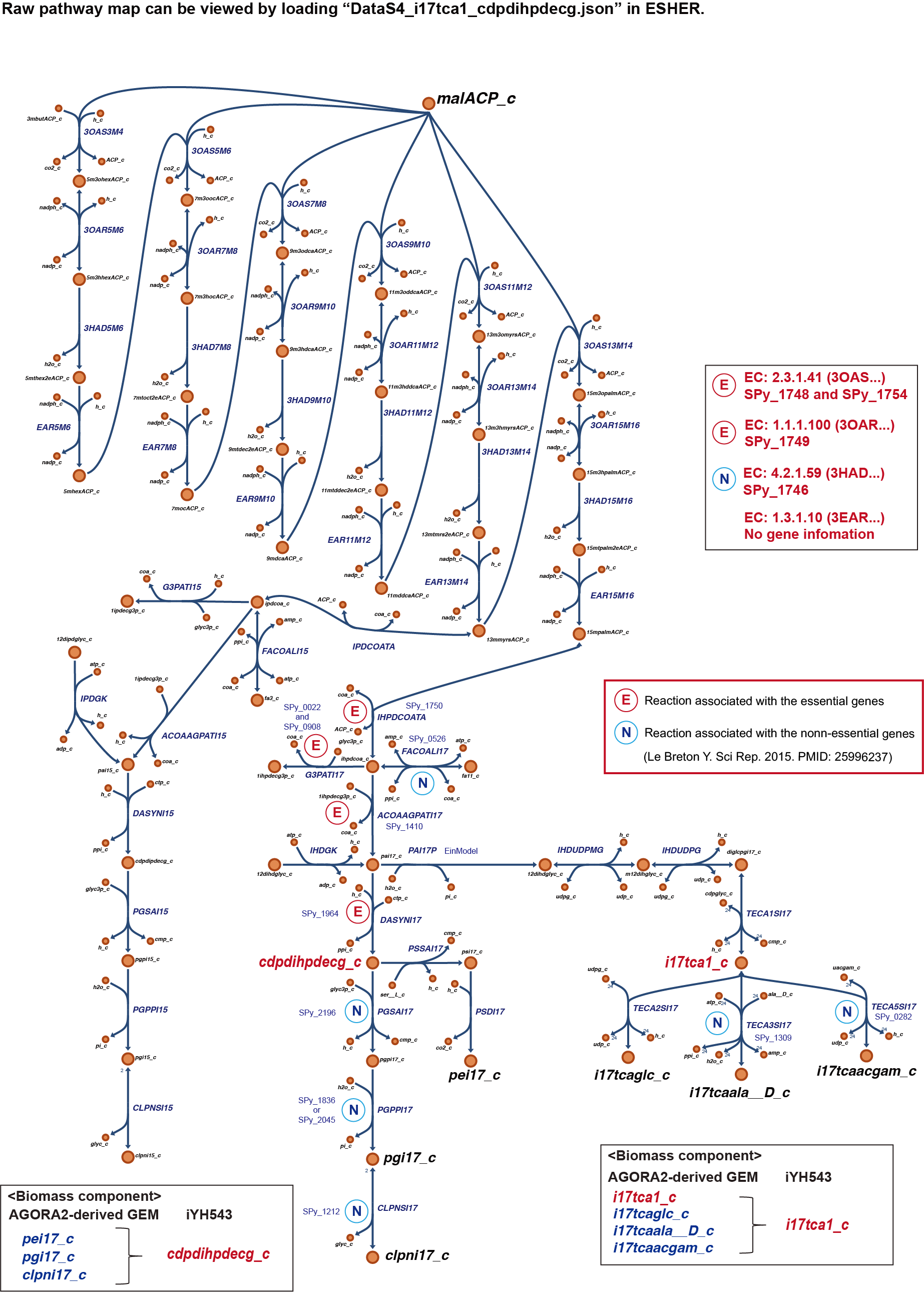
Supplementary Figure 3. The modification of i17tca1_c- and cdpdihpdecg_c- related biomass based on the gene essentiality.**

**
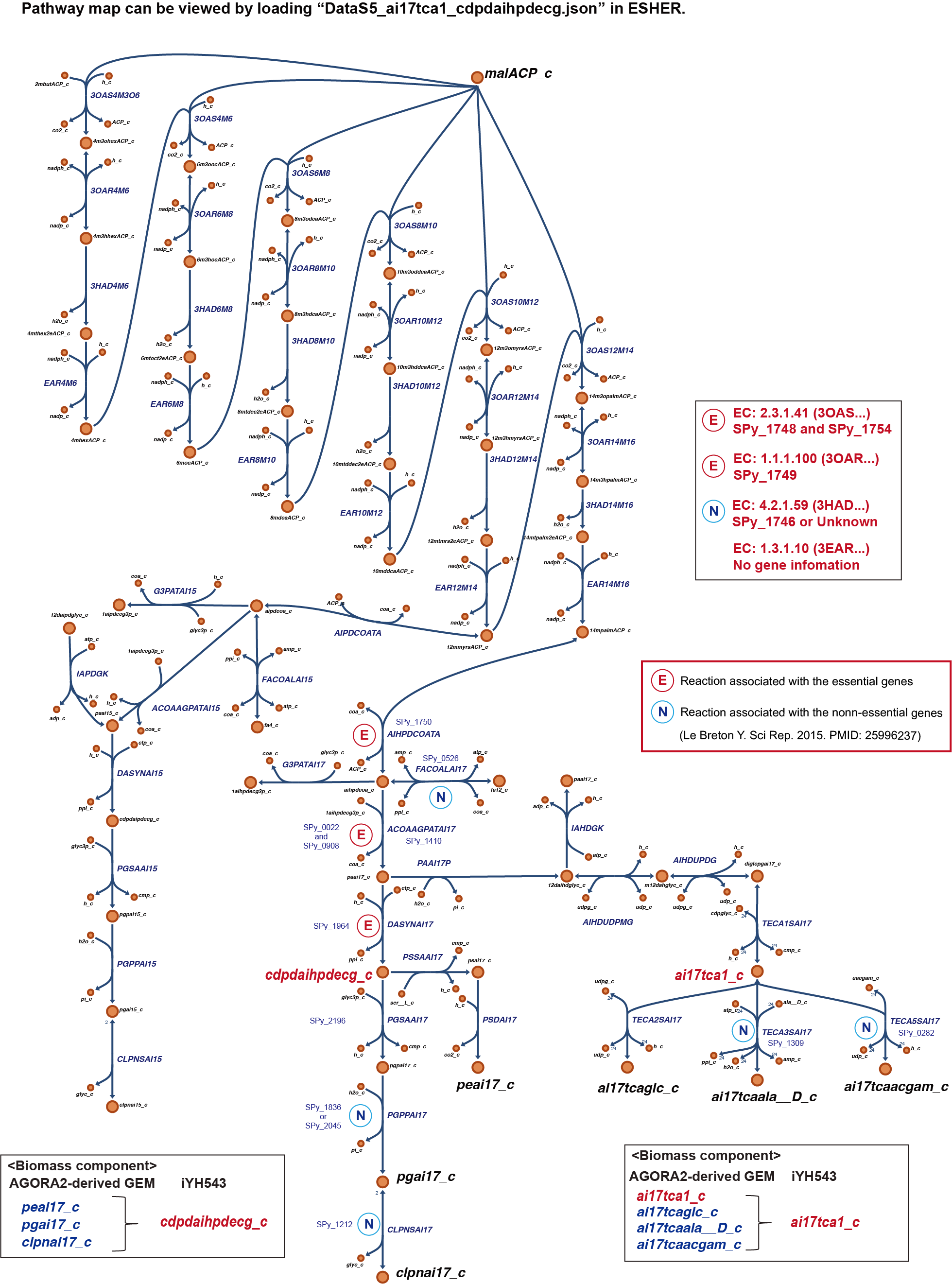
**

**Supplementary Figure 4. The modification of ai17tca1_c- and cdpdaihpdecg_c - related biomass based on the gene essentiality.**

**
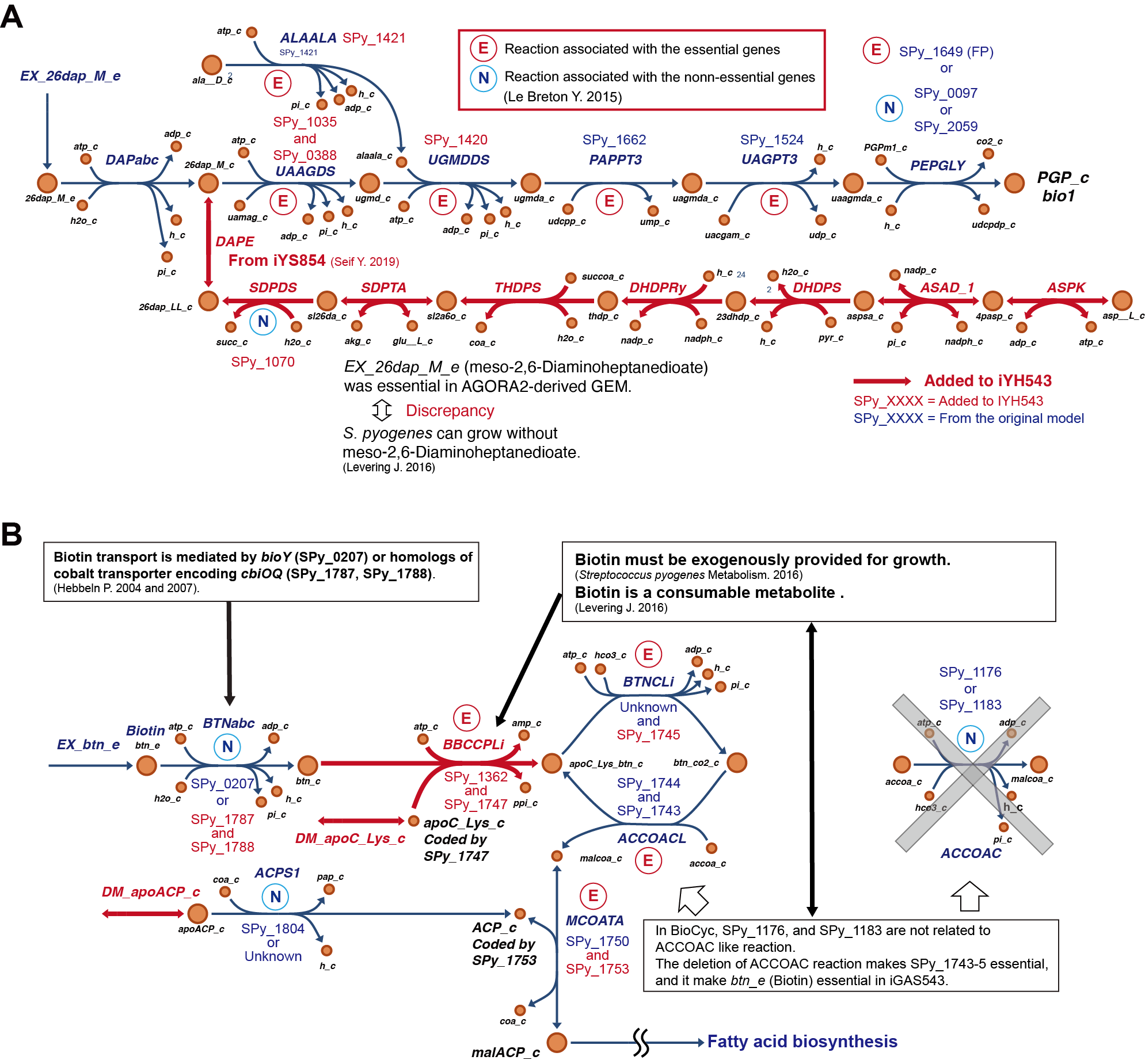
Supplementary Figure 5. Significantly modified metabolic pathways.**

(A) Meso-2,6-Diaminoheptanedioate synthesis pathway from L-aspartate.

(B) The pathway to synthesize lipids from biotin.

All reactions, metabolites, and gene–protein–reaction (GPR) rules in iYH543 are detailed in Supplementary Table 1 and can be found in json format in Supplementary Data 1.

bio1: a component of biomass (a reactant in formula for biomass, bio1 reaction).

Gene essentiality (Le Breton. 2015)[5] (PMID: 25996237)

CDM2 components and about the consumption of biotin (Levering J. 2016)[1] (PMID: 26970054)

iYS854, GEM for *Staphylococcus aureus* (Seif Y. 2019)[6, 7] (PMID: 30625152)

The evidences for biotin transporter (Hebbeln P. 2004 and 2007)[8] (PMID: 17301237, PMID: 14734175).

About the essentialities for biotin (*Streptococcus pyogenes* Metabolism)[9] (PMID: 26866220).


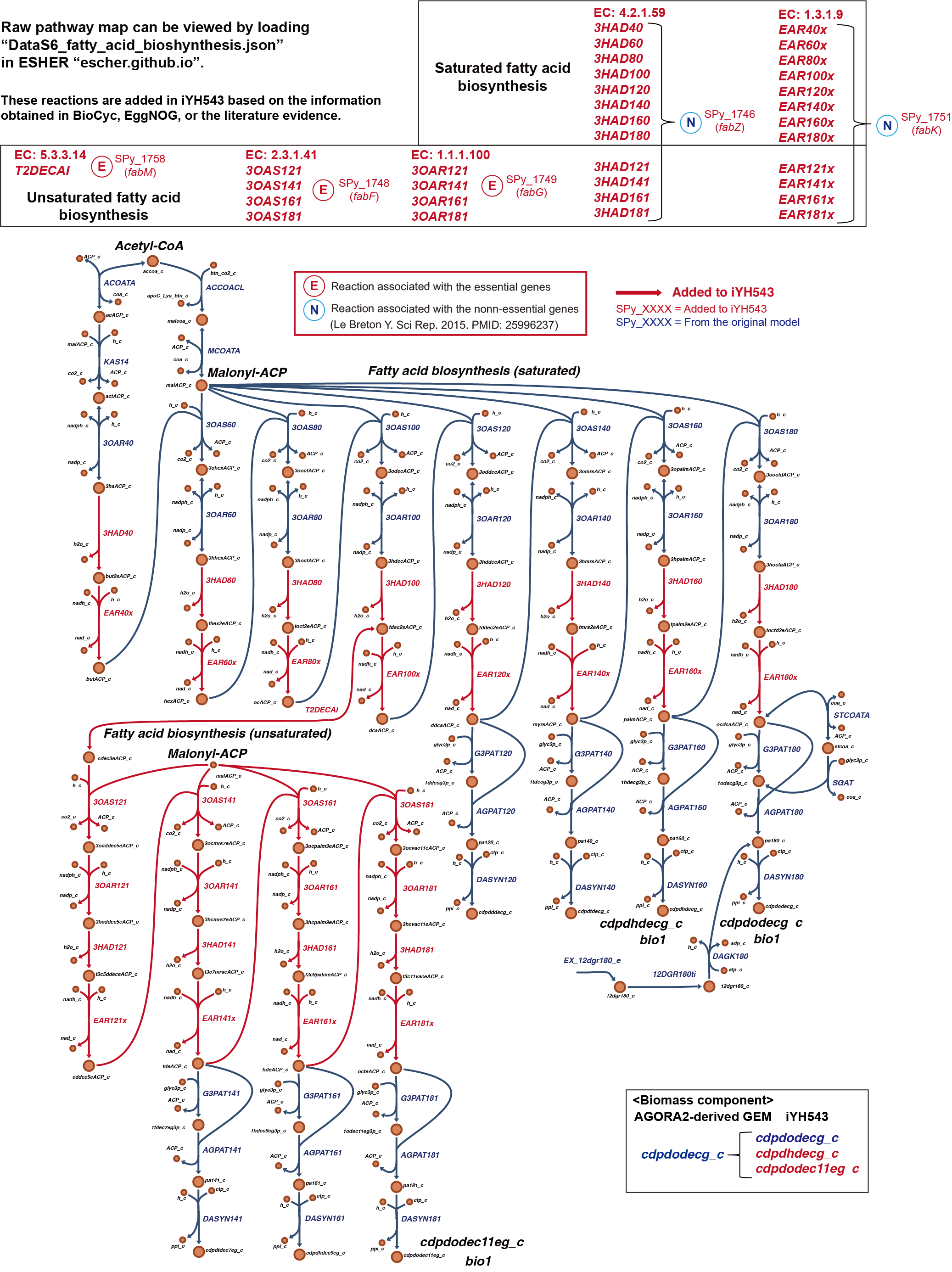


**Supplementary Figure 6. Fatty acid biosynthesis pathway in iYH543.**

**
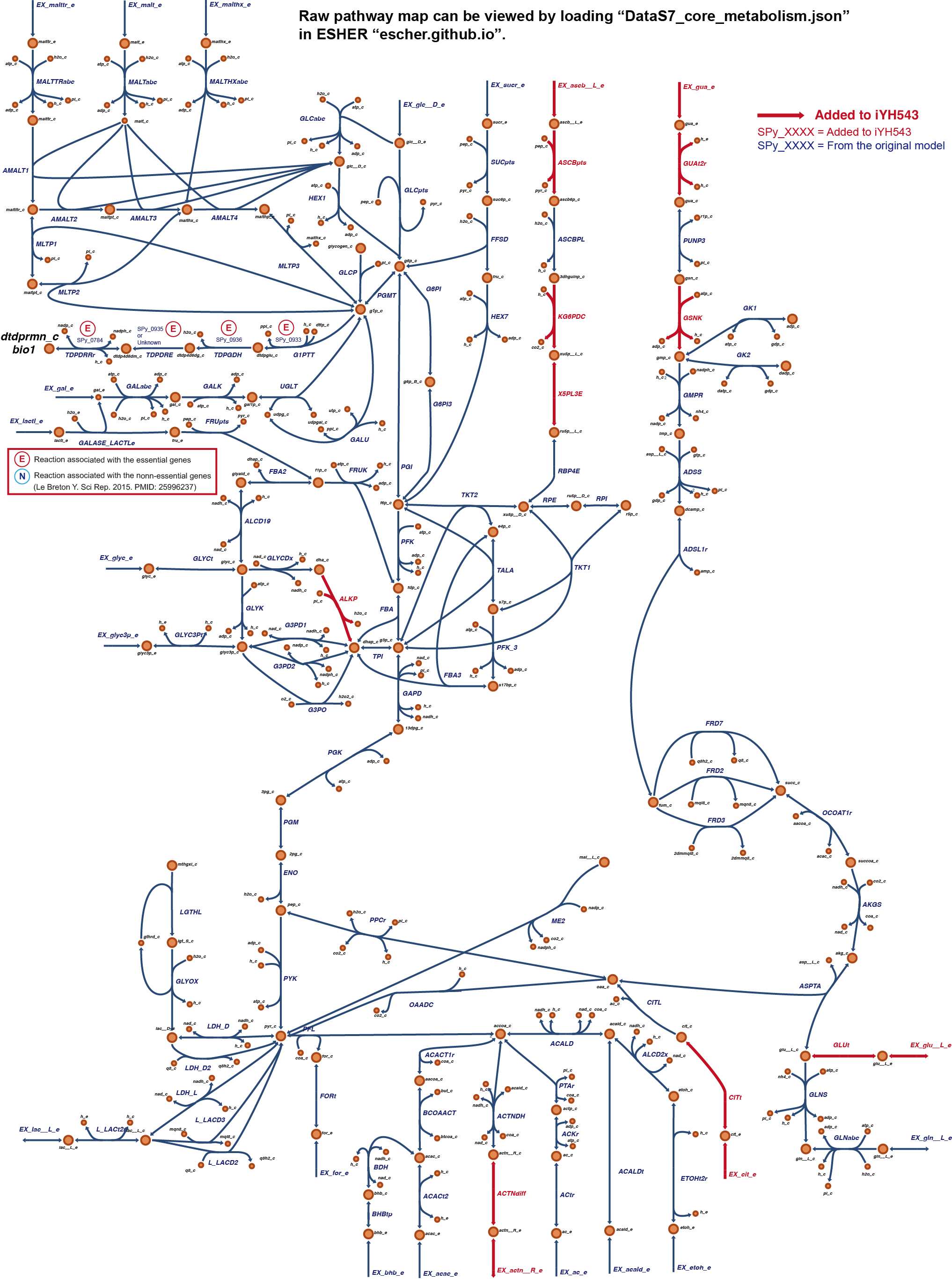
**

**Supplementary Figure 7. Core metabolism in iYH543.**

**
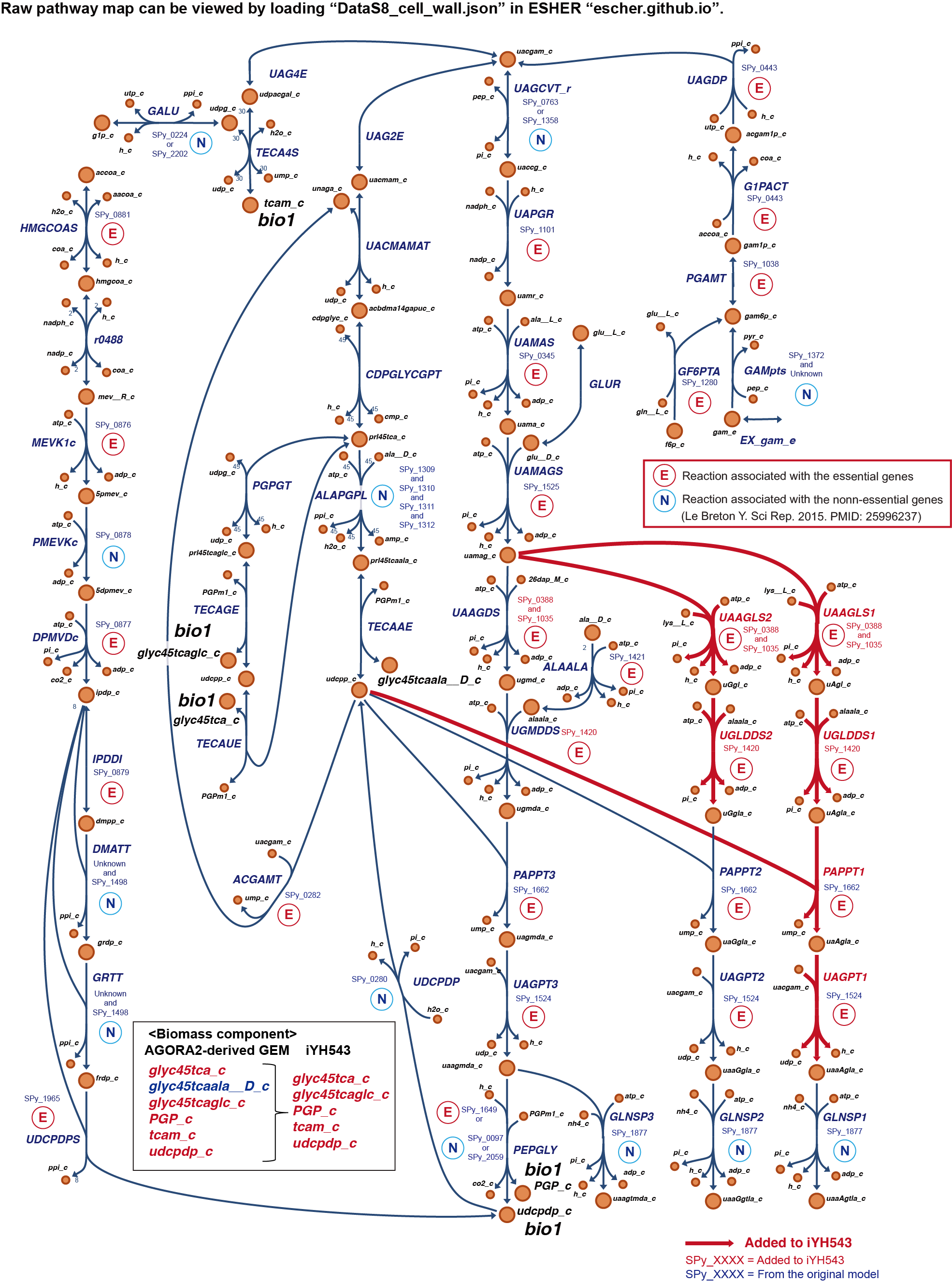
**

**Supplementary Figure 8. Cell wall synthesis pathway in iYH543.**

**
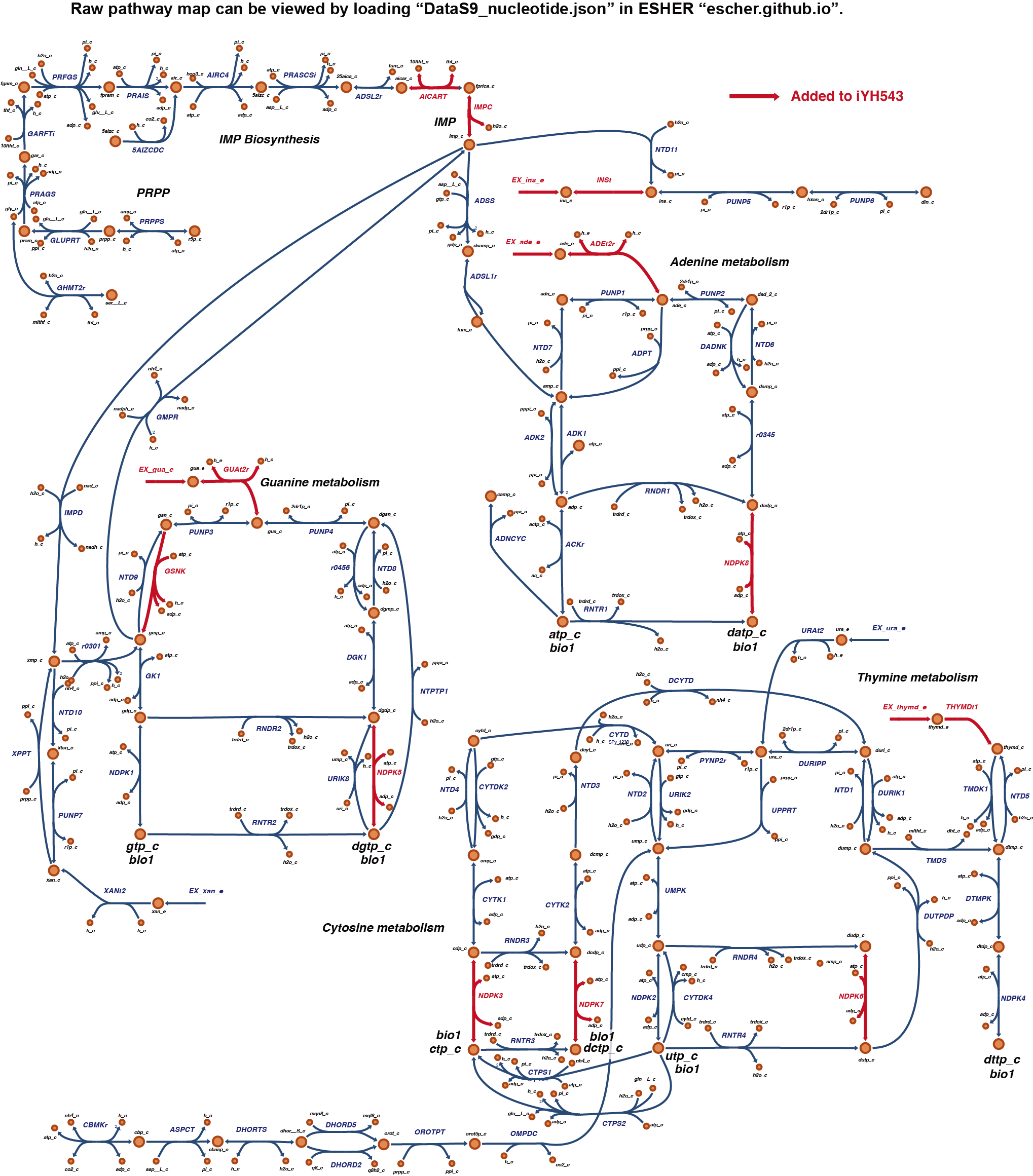
**

**Supplementary Figure 9. Nucleotide metabolism in iYH543.**


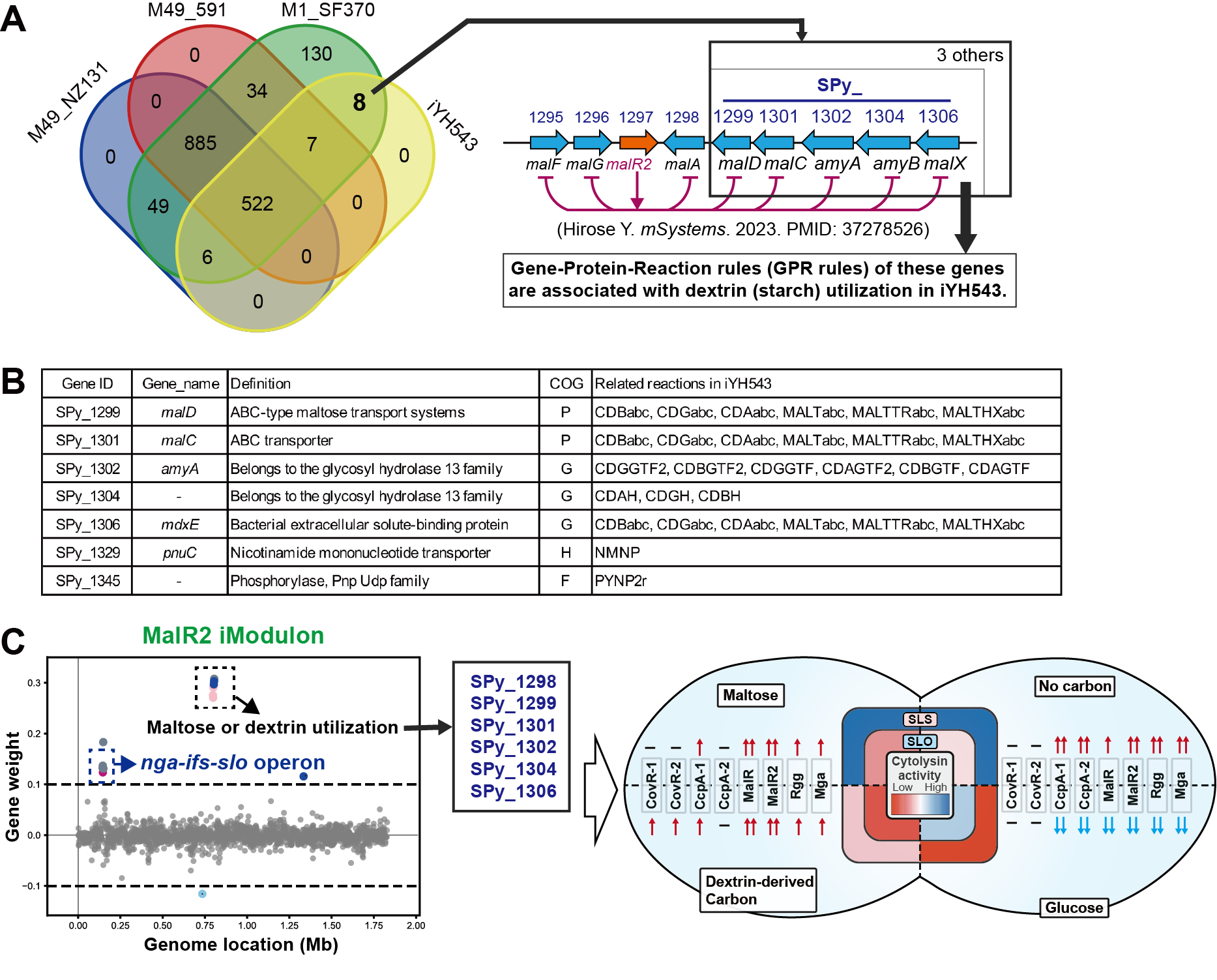


**Supplementary Figure 10. The similarity and difference of genomes between M49 strains and M1 SF370.**

(A) Venn diagram to illustrate the shared genes between serotype M49 strains and M1 SF370. Blue indicates 1463 genes of M49 NZ131, which have the homolog against M1 SF 370 genes. Red indicates 1448 genes of M49 591, which have the homolog against M1 SF 370 genes. Green indicates all 1641 genes of M1 SF370. Yellow indicates all 543 genes in iYH543.

(B) Eight genes M49 strains do not have in iYH543.

(C) Gene weights in MalR2 iModulon of *S. pyogenes* (<https://imodulondb.org/>), which allowed us to reveals carbon sources that control bacterial expression of hemolytic toxins (Hirose Y. 2023)[10](PMID: 37278526). Maltose is a disaccharide of D-glucose, whereas dextrin is a polysaccharide of D-glucose. Despite this structural similarity, these carbon sources induced different hemolytic activity in *S. pyogenes*. The mechanism for the change in hemolytic toxin expression according to the carbon sources was unknown. SLS, Streptolysin S. SLO, Streptolysin O.

**References**

[1] Levering J, Fiedler T, Sieg A, van Grinsven KW, Hering S, Veith N, et al. Genome-scale reconstruction of the *Streptococcus pyogenes* M49 metabolic network reveals growth requirements and indicates potential drug targets. J Biotechnol. 2016;232:25-37.

[2] Tang L, Wang W, Zhou W, Cheng K, Yang Y, Liu M, et al. Three-pathway combination for glutathione biosynthesis in Saccharomyces cerevisiae. Microb Cell Fact. 2015;14:139.

[3] Fan C, Moews PC, Shi Y, Walsh CT, Knox JR. A common fold for peptide synthetases cleaving ATP to ADP: glutathione synthetase and D-alanine:d-alanine ligase of *Escherichia coli*. Proc Natl Acad Sci U S A. 1995;92:1172-6.

[4] Janowiak BE, Griffith OW. Glutathione synthesis in *Streptococcus agalactiae*. One protein accounts for gamma-glutamylcysteine synthetase and glutathione synthetase activities. J Biol Chem. 2005;280:11829-39.

[5] Le Breton Y, Belew AT, Valdes KM, Islam E, Curry P, Tettelin H, et al. Essential Genes in the Core Genome of the Human Pathogen *Streptococcus pyogenes*. Sci Rep. 2015;5:9838.

[6] Seif Y, Monk JM, Mih N, Tsunemoto H, Poudel S, Zuniga C, et al. A computational knowledge-base elucidates the response of *Staphylococcus aureus* to different media types. PLoS Comput Biol. 2019;15:e1006644.

[7] Hebbeln P, Eitinger T. Heterologous production and characterization of bacterial nickel/cobalt permeases. FEMS Microbiol Lett. 2004;230:129-35.

[8] Hebbeln P, Rodionov DA, Alfandega A, Eitinger T. Biotin uptake in prokaryotes by solute transporters with an optional ATP-binding cassette-containing module. Proc Natl Acad Sci U S A. 2007;104:2909-14.

[9] Pancholi V, Caparon M. *Streptococcus pyogenes* Metabolism. In: Ferretti JJ, Stevens DL, Fischetti VA, editors. *Streptococcus pyogenes* : Basic Biology to Clinical Manifestations. Oklahoma City (OK)2016.

[10] Hirose Y, Poudel S, Sastry AV, Rychel K, Lamoureux CR, Szubin R, et al. Elucidation of independently modulated genes in Streptococcus pyogenes reveals carbon sources that control its expression of hemolytic toxins. mSystems. 2023;8:e0024723.
